# [^18^F]FDG PET/CT detection and therapeutic response assessment of primary multisystem extranodal diffuse large B-cell lymphoma without lymph node involvement: a case report

**DOI:** 10.3389/fonc.2025.1697944

**Published:** 2025-12-15

**Authors:** Taiping Liao, Huan Deng, Yongjun Long, Jun He, Lingxiao Li, Cong Xiao, Jun Li

**Affiliations:** 1 Department of Nuclear Medicine, The Third Hospital of Mianyang (Sichuan Mental Health Center), Mianyang, China; 2 School of Clinical Medicine, North Sichuan Medical College, Nanchong, China; 3 Department of Oncology, The Third Hospital of Mianyang (Sichuan Mental Health Center), Mianyang, China; 4 Department of Orthopedics, The Third Hospital of Mianyang(Sichuan Mental Health Center), Mianyang, China

**Keywords:** [18F]FDG, case report, diffuse large B-cell lymphoma, extranodal, PET/CT

## Abstract

**Introduction:**

Primary extranodal diffuse large B-cell lymphoma (PE-DLBCL) originates in extranodal organs and accounts for approximately 30%–40% of all DLBCL cases. PE-DLBCL involving multiple organs without lymph node involvement is uncommon. We present a case of DLBCL in a patient who initially presented with abdominal pain. Abdominal computed tomography (CT) revealed suspicious neoplastic lesions in the liver and ileum. Histopathological examination of an ileal biopsy confirmed the diagnosis of DLBCL. The patient subsequently underwent [^18^F]FDG PET/CT for disease staging. PET/CT demonstrated multiple hypermetabolic lesions in the liver, right kidney, and ileum, notably in the absence of any lymph node involvement throughout the body. After four cycles of R-CHOP (rituximab, cyclophosphamide, doxorubicin, vincristine, and prednisone) chemotherapy, [^18^F]FDG PET/CT evaluation revealed significant shrinkage of the abdominal lesions, indicating a favorable prognosis.

**Conclusion:**

This case demonstrates a rare presentation of multisystem PE-DLBCL without lymphadenopathy and underscores the vital role of [^18^F]FDG PET/CT in identifying disease extent and guiding accurate staging and management, as well as evaluating treatment response.

## Introduction

DLBCL is the most common subtype of non-Hodgkin lymphoma, characterized by highly aggressive behavior and heterogeneous clinical manifestations, which pose significant diagnostic and therapeutic challenges (1). While most DLBCL cases primarily involve the lymph nodes, a subset originates in extranodal organs. The gastrointestinal tract is the most common primary site of PE-DLBCL (2), while primary extranodal disease may also arise in the skin (3), breast (4), central nervous system (5), and other locations (6).

According to DLBCL clinical guidelines and SEER database analyses, PE-DLBCL accounts for approximately 30%-40% of all DLBCL cases (7, 8). Large-cohort epidemiological studies from multiple regions also provide important insights into the distribution of extranodal DLBCL (EN-DLBCL). Babu SM et al. (9) reported 526 DLBCL cases in India, among which 202 were EN-DLBCL. Economopoulos T et al. (10) analyzed 810 Non-Hodgkin Lymphoma (NHL) cases in Greece and identified 23 cases of multiple EN-DLBCL. Candelaria M et al. (11) reported 51 PE-DLBCL cases among 637 DLBCL patients in Mexico. More recently, Chen SY et al. (12) conducted a large Chinese cohort study involving 4,785 EN-DLBCL cases, including 3,163 single-organ and 1,621 multiple-organ presentations. As shown in **Table 1**.

**Table 1:** Overview of epidemiological cohort studies on EN-DLBCL.

Author	Data source	Year	Total sample size	Number of EN-DLBCL cases
Gupta V (8)	USA	2022	DLBCL:93638	EN-DLBCL:32284
Babu SM (9)	India	2018	DLBCL: 526	EN-DLBCL:202
Economopoulos T (10)	Greece	2005	NHL: 810	Multiple EN-DLBCL:23
Candelaria M (11)	Mexico	2019	DLBCL:637	PE-DLBCL:51
Chen SY (12)	China	2025	EN-DLBCL:4785	Single EN-DLBCL:3163 Multiple EN-DLBCL:1621

DLBCL, Diffuse Large B-Cell Lymphoma; EN-DLBCL,extranodal diffuse large B-cell lymphoma; PE-DLBCL, primary extranodal diffuse large B-cell lymphoma; NHL, Non-Hodgkin Lymphoma; SEER, Surveillance, Epidemiology, and End Results Program.

PE-DLBCL should be distinguished from extranodal involvement of DLBCL (ENI-DLBCL), as the former refers to lymphoma originating in an extranodal organ, whereas the latter represents secondary extension of nodal disease into extranodal sites. Involvement of multiple extranodal organs can mimic metastatic carcinoma, potentially leading to diagnostic challenges, and definitive diagnosis often relies on histopathological confirmation through biopsy. Due to the low incidence and lack of large-scale studies, the clinical behavior and prognostic characteristics of such cases remain unclear. Most reports in the literature are individual case studies; for instance, some researchers have described cases of primary extranodal lymphoma predominantly involving the kidneys (13, 14). In these cases, initial staging was performed using [^18^F]FDG PET/CT prior to chemotherapy, followed by post-treatment PET/CT for response evaluation. The lesions showed marked reduction in size and metabolic activity, indicating a significant therapeutic response.

[^18^F]FDG PET/CT demonstrates high sensitivity for detecting hypermetabolic lesions and is particularly valuable in assessing the extent of disease in atypical cases with extranodal involvement. It plays a crucial role in the staging and treatment response evaluation of extranodal lymphomas (15). Moreover, semiquantitative PET/CT parameters, such as metabolic tumor volume (MTV) and total lesion glycolysis (TLG), may provide useful information for risk stratification and prognostic assessment (16, 17), thereby offering essential guidance for treatment decision-making.

Here, we present a case of DLBCL with simultaneous involvement of multiple extranodal organs without lymph node infiltration, as identified by [^18^F]FDG PET/CT. This case further highlights the diagnostic precision of PET/CT in DLBCL and its substantial value in guiding clinical decision-making.

## Case presentation

**Figure 1:**
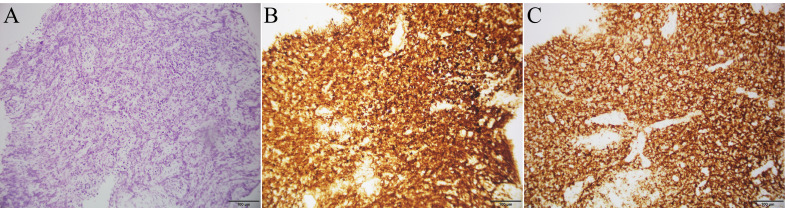
Histopathological and immunohistochemical findings of ileal biopsy: **(A)** Histopathological image of ileal tissue stained with hematoxylin and eosin; **(B)** Immunohistochemical image of Bcl-2; **(C)** Immunohistochemical image of CD20.

**Figure 2:**
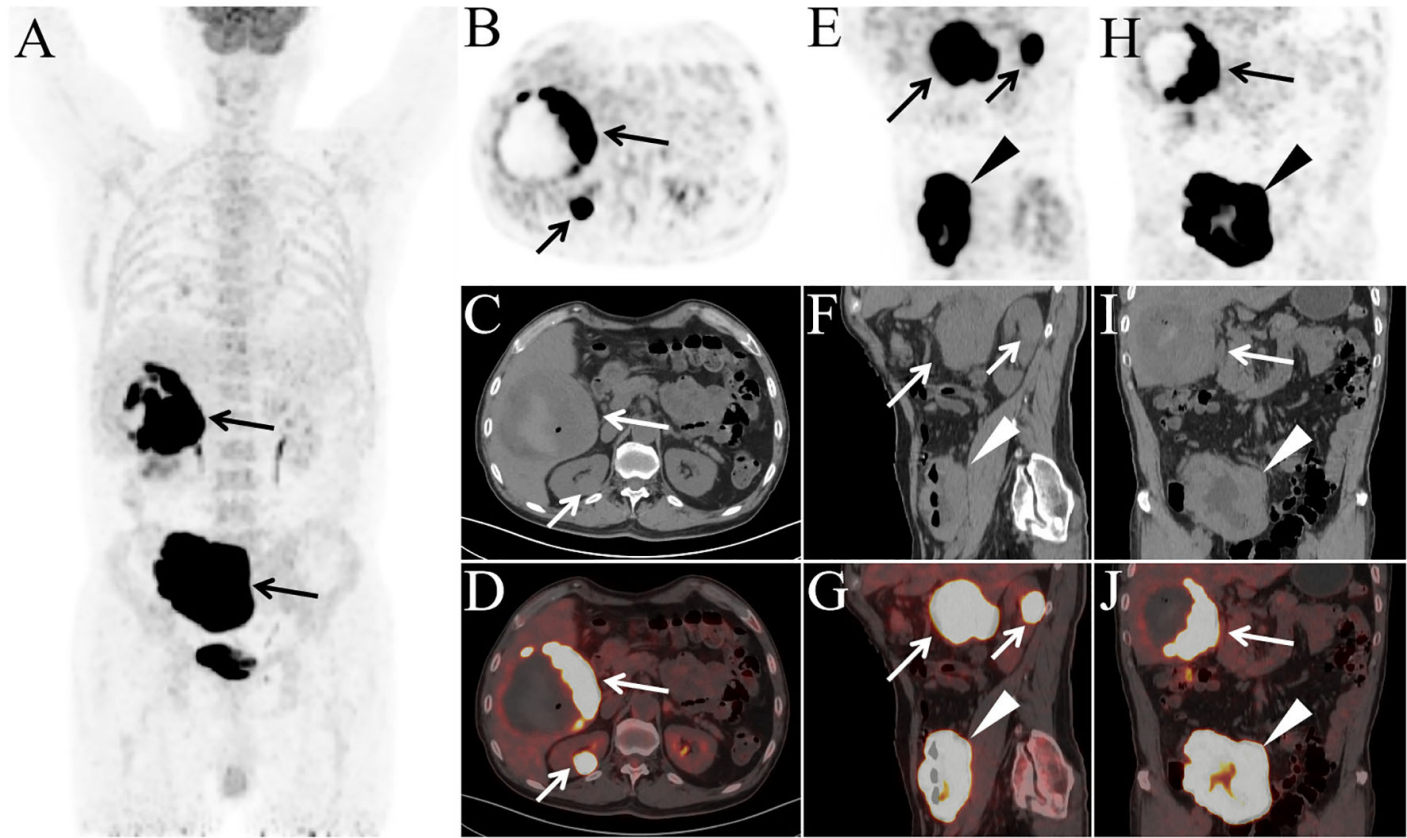
The patient underwent PET/CT examination for staging [**(A)** MIP; **(B, E, H)** PET; **(C, F, I)** CT; **(D, G, J)** PET/CT]. MIP image shows abnormally increased FDG uptake in the right upper and lower abdominal regions (A, arrows). Axial **(B–D)**, sagittal **(E–G),** and coronal **(H–J)** PET/CT images demonstrate a large mass in the posterior segment of the right hepatic lobe measuring 11.0 cm × 10.2 cm × 10.5 cm (B–J, long arrows), a slightly hyperdense nodule in the right kidney measuring 2.6 cm × 2.2 cm × 2.6 cm (B–G, short arrows), and diffuse thickening of the ileal wall with a maximum wall thickness of 4.3 cm (E–J, arrowheads), all exhibiting markedly elevated FDG uptake (SUVmax: hepatic lesion, 32.3; renal lesion, 34.1; ileal lesion, 34.3). Central necrosis, hemorrhage, and a small amount of intralesional gas were observed within the hepatic mass. Notably, there was no evidence of lymphadenopathy or abnormal FDG uptake in any lymph nodes throughout the body.

**Figure 3:**
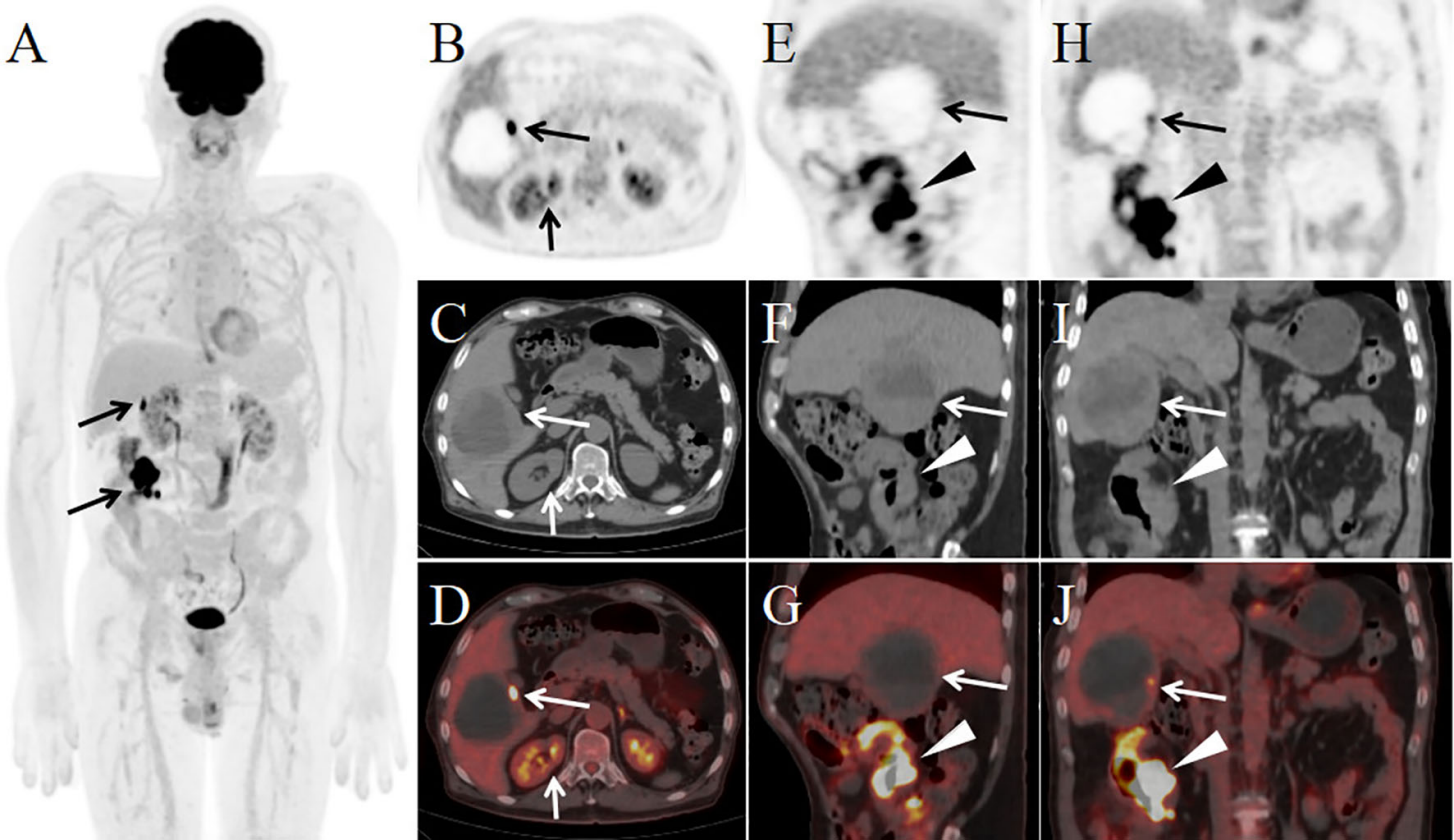
PET/CT images after four cycles of chemotherapy [**(A)** MIP; **(B, E, H)** PET; **(C, F, I)** CT; **(D, G, J)** PET/CT]. The MIP image **(A)** reveals nodular and patchy areas of abnormally increased FDG uptake in the liver and right abdomen. Axial **(B–D)**, sagittal **(E–G)**, and coronal **(H–J)** PET/CT images demonstrate a hepatic lesion (B–J, long arrows) measuring 7.3 cm × 7.6 cm × 7.9 cm, with nodular areas of increased FDG uptake at the lesion margins (SUVmax: 13.9), while FDG uptake is absent in the remaining portions of the lesion. The ileal lesion **(E–J,** arrowheads**)** shows a markedly reduced extent compared to baseline, with a maximum wall thickness of 2.1 cm and abnormally increased FDG uptake (SUVmax: 21.2). The previously noted right renal lesion **(B–D,** short arrows**)** has completely resolved, with no abnormal FDG uptake observed.

A 78-year-old male presented with abdominal pain and underwent contrast-enhanced CT at an outside hospital, which revealed a large hepatic mass with internal hemorrhage and significant thickening of the ileal wall, both highly suspicious for malignancy. The patient exhibited no B symptoms such as fever, night sweats, or weight loss, and routine laboratory tests, including blood counts, liver and renal function, electrolytes, and tumor markers, were unremarkable. The patient had no significant past medical history and no family history of malignancy. The patient initially received interventional treatment to control hepatic bleeding. After achieving hemodynamic stabilization, an ileal biopsy was performed via colonoscopy, and pathological examination confirmed the diagnosis of diffuse large B-cell lymphoma (**Figure 1A**). Immunohistochemical staining (**Figures 1B, C**) showed the following profile: CD20(+), CD79α(+), CD3 (scattered+), CD45RO (scattered+), CD5 (scattered+), CD23(−), Bcl-2 (90%+), Bcl-6 (90%+), C-MYC (50%+), CD10 (+), MUM-1(+), CD56(−), TIA-1(−), P-CK(−), and Ki-67 (90%+). Immunohistochemical analysis revealed positivity for CD20 and CD79α, confirming B-cell lineage. High expression levels of Bcl-2, Bcl-6, and C-MYC, along with a Ki-67 index of 90%, indicated high tumor aggressiveness. The CD10(+)/MUM-1(+) profile suggested a germinal center B-cell (GCB) subtype, supporting the histopathological diagnosis of DLBCL. For disease staging, the patient was referred to our hospital for [^18^F]FDG PET/CT. [^18^F]FDG was administered intravenously at a dose of approximately 5.5 MBq/kg, and imaging was performed using a United Imaging uMI 550 PET/CT system. PET/CT (**Figures 2A–J**) revealed markedly increased FDG uptake in both hepatic and ileal lesions. Additionally, PET/CT identified a slightly hyperdense nodule in the right kidney—missed on prior CT—with significantly elevated FDG metabolism. The patient underwent biopsies of both the liver and kidney lesions at another hospital prior to receiving further treatment, and the pathological results confirmed the diagnosis of lymphoma. This effectively ruled out the possibility of multiple primary malignancies.The final stage of lymphoma was determined to be stage IV, with an International Prognostic Index(IPI) score of 3. Three months later, the patient completed four cycles of R-CHOP chemotherapy. Follow-up [^18^F]FDG PET/CT (**Figures 3A–J**) demonstrated significant reduction in the size of hepatic, ileal, and renal lesions with markedly decreased FDG metabolism, indicating an excellent treatment response. The patient's disease timeline is shown in **Table 2**.

**Table 2:** Timeline of clinical presentation and management.

Time	Event
2024/12/16	Abdominal pain → Contrast-enhanced CT → Interventional procedure to control bleeding from hepatic mass
2025/01/02	Colonoscopy and ileal biopsy → Pathological diagnosis of DLBCL
2025/01/06	[^18^F]FDG PET/CT: Initial staging
2025/01/10-2025/04/10	Four cycles of R-CHOP chemotherapy
2025/04/15	[^18^F]FDG PET/CT: Treatment Response Evaluation
2025/04/20	Telephone follow-up: Significant clinical improvement
2025/01/10-2025/07/10	Eight cycles of R-CHOP chemotherapy
2025/11/15	Telephone follow-up: The patient did not report any specific discomfort

## Discussion

Diffuse large B-cell lymphoma (DLBCL) is the most prevalent subtype of non-Hodgkin lymphoma. In this case, although initial CT scans revealed hepatic and ileal abnormalities, the renal lesion was missed due to its small size and subtle density changes. [^18^F]FDG PET/CT, owing to its superior sensitivity in detecting lymphoma involvement, successfully identified focal hypermetabolism in the renal cortex, thereby improving staging accuracy. This observation aligns with findings from Le Dortz L et al., who emphasized the role of PET/CT in refining lymphoma staging and avoiding both under- and overtreatment (18). Notably, a maximum standardized uptake value (SUVmax) greater than 10 is suggestive of high-grade or aggressive lymphoma (19). In this patient, SUVmax values exceeded 30 in all three involved organs—liver, kidney, and ileum—indicating high tumor aggressiveness. These metabolic findings correlate with the pathological results, particularly the high expression levels of Bcl-2, Bcl-6, and C-MYC (20), reinforcing the utility of SUVmax in assessing lymphoma aggressiveness.Reports of multisystem extranodal lymphoma are rare, and most involve some degree of lymph node infiltration (21, 22). Cases with a complete absence of lymphadenopathy are exceptionally uncommon (23). This case highlights a unique presentation of DLBCL with simultaneous involvement of the liver, kidney, and ileum, without any lymph node involvement. It further underscores the indispensable role of [^18^F]FDG PET/CT in the accurate staging of lymphoma. At a three-month follow-up conducted via telephone, the patient reported good overall health, no B symptoms, and stable general condition. [^18^F]FDG PET/CT demonstrated a marked reduction in the size of hepatic, renal, and ileal lesions, indicating a favorable treatment response and prognosis. At a ten-month telephone follow-up, the patient had completed eight cycles of chemotherapy and did not report any specific discomfort.

## Data Availability

The original contributions presented in the study are included in the article/**Supplementary Material**. Further inquiries can be directed to the corresponding authors.
