# Supplementary material for: [18F]FDG PET/CT detection and therapeutic response assessment of primary multisystem extranodal diffuse large B-cell lymphoma without lymph node involvement: a case report
Source: Front Oncol. 2025 Dec 15;15:1697944. doi: 10.3389/fonc.2025.1697944 (PMC12745402; doi:10.3389/fonc.2025.1697944)
Supplement: Supplementary file 1 [file DataSheet1.pdf]

Eur J Nucl Med Mol Imaging (2015) 42:328–354
DOI 10.1007/s00259-014-2961-x

GUIDELINES

FDG PET/CT: EANM procedure guidelines for tumour imaging: version 2.0

Ronald Boellaard · Roberto Delgado-Bolton · Wim J. G. Oyen · Francesco Giammarile · Klaus Tatsch · Wolfgang Eschner · Fred J. Verzijlbergen · Sally F. Barrington · Lucy C. Pike · Wolfgang A. Weber · Sigrid Stroobants · Dominique Delbeke · Kevin J. Donohoe · Scott Holbrook · Michael M. Graham · Giorgio Testanera · Otto S. Hoekstra · Josee Zijlstra · Eric Visser · Corneline J. Hoekstra · Jan Pruim · Antoon Willemsen · Bertjan Arends · Jörg Kotzerke · Andreas Bockisch · Thomas Beyer · Arturo Chiti · Bernd J. Krause

Received: 10 November 2014 / Accepted: 12 November 2014 / Published online: 2 December 2014
© The Author(s) 2015. This article is published with open access at Springerlink.com

Abstract The purpose of these guidelines is to assist physicians in recommending, performing, interpreting and reporting the results of FDG PET/CT for oncological imaging of adult patients. PET is a quantitative imaging technique and therefore requires a common quality control (QC)/quality assurance (QA) procedure to maintain the accuracy and precision of quantitation. Repeatability and reproducibility are two essential requirements for any quantitative measurement and/or imaging biomarker. Repeatability relates to the uncertainty in obtaining the same result in the same patient when he or she is examined more than once on the same

R. Boellaard (✉) · O. S. Hoekstra
Department of Radiology & Nuclear Medicine, VU University Medical Centre, De Boelelaan 1117, 1081 HV Amsterdam, The Netherlands
e-mail: r.boellaard@vumc.nl

R. Delgado-Bolton
Department of Diagnostic Imaging (Radiology) and Nuclear Medicine, San Pedro Hospital and Centre for Biomedical Research of La Rioja (CIBIR), University of La Rioja, Logroño, La Rioja, Spain

W. J. G. Oyen · E. Visser
Department of Radiology & Nuclear Medicine, Radboud University Nijmegen Medical Centre, Nijmegen, The Netherlands

F. Giammarile
Department of Nuclear Medicine, Centre Hospitalier Universitaire de Lyon, Lyon, France

K. Tatsch
Department of Nuclear Medicine, Municipal Hospital Karlsruhe Inc., Karlsruhe, Germany

W. Eschner
Department of Nuclear Medicine, University of Cologne, Cologne, Germany

F. J. Verzijlbergen
Department of Nuclear Medicine, Erasmus Medical Center, Rotterdam, The Netherlands

S. F. Barrington · L. C. Pike
PET Imaging Centre, St Thomas' Hospital, Division of Imaging Sciences and Biomedical Engineering, King's College London, King's Health Partners, London, UK

W. A. Weber
Department of Radiology, Memorial Sloan Kettering Center, New York, NY, USA

S. Stroobants
Department of Nuclear Medicine, Antwerp University Hospital, Antwerp, Belgium

D. Delbeke
Department of Radiology and Radiological Sciences, Vanderbilt University Medical Center, Nashville, TN, USA

K. J. Donohoe
Beth Israel Deaconess Medical Center, Boston, MA, USA

S. Holbrook
Invivo Molecular Imaging LLC, Gray, TN, USA

M. M. Graham
Department of Radiology, University of Iowa, Iowa City, IA, USA

G. Testanera · A. Chiti
Department of Nuclear Medicine, Humanitas Clinical and Research Center, Rozzano, MI, Italy

system. However, imaging biomarkers should also have adequate reproducibility, i.e. the ability to yield the same result in the same patient when that patient is examined on different systems and at different imaging sites. Adequate repeatability and reproducibility are essential for the clinical management of patients and the use of FDG PET/CT within multicentre trials. A common standardised imaging procedure will help promote the appropriate use of FDG PET/CT imaging and increase the value of publications and, therefore, their contribution to evidence-based medicine. Moreover, consistency in numerical values between platforms and institutes that acquire the data will potentially enhance the role of semiquantitative and quantitative image interpretation. Precision and accuracy are additionally important as FDG PET/CT is used to evaluate tumour response as well as for diagnosis, prognosis and staging. Therefore both the previous and these new guidelines specifically aim to achieve standardised uptake value harmonisation in multicentre settings.

Keywords FDG · PET/CT · Imaging procedure · Tumour · Oncology · Quantification

J. Zijlstra
Department of Hematology, VU University Medical Centre, Amsterdam, The Netherlands

C. J. Hoekstra
Department of Nuclear Medicine, Jeroen Bosch Hospital, Den Bosch, The Netherlands

J. Pruim · A. Willemsen
Department of Nuclear Medicine & Molecular Imaging, University Medical Centre Groningen, Groningen, The Netherlands

B. Arends
Department of Clinical Physics, Catharina Hospital, Eindhoven, The Netherlands

J. Kotzerke
Clinic and Outpatient Clinic for Nuclear Medicine, University Hospital Dresden, Dresden, Germany

A. Bockisch
Clinic for Nuclear Medicine, University Hospital Essen, Essen, Germany

T. Beyer
Centre for Medical Physics and Biomedical Engineering, Medical University of Vienna, Vienna, Austria

B. J. Krause
Department of Nuclear Medicine, University Hospital Rostock, Rostock, Germany

Preamble

The European Association of Nuclear Medicine (EANM) is a professional nonprofit medical association that facilitates communication worldwide among individuals pursuing clinical and research excellence in nuclear medicine. The EANM was founded in 1985.

These guidelines are intended to assist practitioners in providing appropriate nuclear medicine care for patients. They are not inflexible rules or requirements of practice and are not intended, nor should they be used, to establish a legal standard of care.

The ultimate judgment regarding the propriety of any specific procedure or course of action must be made by medical professionals taking into account the unique circumstances of each case. Thus, there is no implication that an approach differing from the guidelines, standing alone, is below the standard of care. To the contrary, a conscientious practitioner may responsibly adopt a course of action different from that set out in the guidelines when, in the reasonable judgment of the practitioner, such course of action is indicated by the condition of the patient, limitations of available resources or advances in knowledge or technology subsequent to publication of the guidelines.

The practice of medicine involves not only the science but also the art of dealing with the prevention, diagnosis, alleviation and treatment of disease. The variety and complexity of human conditions make it impossible to always reach the most appropriate diagnosis or to predict with certainty a particular response to treatment. Therefore, it should be recognised that adherence to these guidelines will not ensure an accurate diagnosis or a successful outcome. All that should be expected is that the practitioner will follow a reasonable course of action based on current knowledge, available resources and the needs of the patient to deliver effective and safe medical care. The sole purpose of these guidelines is to assist practitioners in achieving this objective.

Introduction

^{18}F-FDG (FDG) PET imaging is a noninvasive diagnostic tool that provides tomographic images and can be used to obtain quantitative parameters concerning the metabolic activity of target tissues. ^{18}F is a cyclotron-produced radioisotope of fluorine that emits positrons and has a short half-life (109.7 min). It allows labelling of numerous molecular tracers that can be imaged within a few hours (typically <3 h) after injection. FDG is an analogue of glucose and is taken up by living cells via cell membrane glucose transporters and subsequently incorporated into the first step of the normal glycolytic pathway.

PET is a tomographic technique that measures the three-dimensional distribution of positron-emitting labelled radiotracers. PET allows noninvasive quantitative assessment of

biochemical and functional processes. The most commonly used tracer at present is the ^{18}F-labelled glucose analogue FDG. FDG accumulation in tissue is proportional to the amount of glucose utilisation. Increased consumption of glucose is characteristic of most cancers and is in part related to overexpression of the GLUT glucose transporters and increased hexokinase activity. FDG PET has been proven to be a sensitive imaging modality for detection, staging and restaging and therapy response assessment in oncology [1–13]. FDG PET/CT provides essential information for radiation treatment planning, helping with critical decisions when delineating tumour volumes [14, 15].

CT uses a combined X-ray transmission source and detector system rotating around the subject to generate tomographic images. CT allows not only attenuation correction but also the visualisation of morphological and anatomical structures with a high spatial resolution. Anatomical and morphological information derived from CT can be used to improve the localisation, extent and characterisation of lesions detected by FDG PET. These guidelines focus on the use of FDG PET/CT in oncology, where PET/CT continues to gain importance. Recently, combined or integrated PET and MRI systems (PET/MRI) have come onto the market. PET/MRI technology is, however, still in development and is not yet widely available [16, 17]. Therefore, this version of the guidelines does not address FDG PET/MRI, although currently the quantitative performance of FDG PET/MRI is being explored as a scientific project within EANM Research Limited (EARL).

Goals

The purpose of these guidelines is to assist physicians in recommending, performing, interpreting and reporting the results of FDG PET/CT for oncological imaging of adult and paediatric patients. PET is a quantitative imaging technique and therefore requires a common quality control (QC)/quality assurance (QA) procedure to maintain the accuracy and precision of quantitation [18]. Repeatability and reproducibility are two essential requirements for any quantitative measurement and/or imaging biomarker. Repeatability relates to the uncertainty in obtaining the same result in the same patient when he or she is examined more than once on the same system. However, imaging biomarkers should also have adequate reproducibility, i.e. the ability to yield the same result in the same patient when that patient is examined on different systems and at different imaging sites. Adequate repeatability and reproducibility are essential for the clinical management of patients and the use of FDG PET/CT within multicentre trials. A common standardised imaging procedure will help promote the appropriate use of FDG PET/CT imaging and increase the value of publications and, therefore, their contribution to evidence-based medicine. Moreover, consistency in numerical values between platforms and institutes that acquire the data will potentially enhance the role of semi-quantitative and quantitative image interpretation. Precision and accuracy are additionally important as FDG PET/CT is used to evaluate tumour response as well as for diagnosis, prognosis and staging. Therefore both the previous and these new guidelines specifically aim to achieve standardised uptake value (SUV) harmonisation in multicentre settings.

These guidelines address general information about FDG PET/CT and are provided to help the physician, physicist and technologist perform, interpret and document quantitative FDG PET/CT examinations, but concentrate on harmonisation/standardisation of diagnostic quality and quantitative information in oncology imaging of adult patients. These guidelines present a standardised imaging procedure for static FDG PET/CT data acquisition, QC and QA. Quantification of FDG PET/CT is defined as quantification using SUVs [19] because the SUV represents the most commonly used semiquantitative parameter for analysis of tracer uptake. Furthermore, this new version of the guidelines only addresses combined or integrated whole-body 3D PET/CT systems.

These guidelines build upon the earlier published European procedure guidelines for quantitative FDG PET and PET/CT for tumour imaging [20] and the SNMMI procedure guidelines for tumour imaging with ^{18}F-FDG PET/CT 1.0 [21]. For a detailed history of the document, refer to section History of the document. For FDG PET/CT studies in paediatric patients, refer to the specific guidelines [22].

Definitions

- An integrated or multimodality PET/CT system is a combination of a PET and a CT system with a single, conjoined patient handling system (table).
- PET/CT allows sequential acquisition of PET and CT portions of the examination with the patient in the same position for both examinations. Both datasets are intrinsically coregistered.
- An FDG PET/CT examination may cover various coaxial imaging ranges; these ranges are described as follows, with different denominations depending on European standard (GL 1.0) or US standard (defined in Current Procedural Terminology 2005):

– *Whole-body imaging*: From the top of the head through the feet (standard for both Europe and the US).
– *Torso imaging*: Base of the skull to mid-thigh. Covers most of the relevant portions of the body in many oncological diseases (standard for both Europe and the US). If indicated, cranially extended torso imaging may also cover the brain in the same scan (from the top of the head to mid-thigh).

– *Limited-area tumour imaging*: For the evaluation of tumour-related changes in a limited portion of the body.
– *Whole-body or torso imaging combined with dedicated brain imaging*: Dedicated brain imaging combined with whole-body or torso imaging from base of skull.

- In PET/CT studies attenuation correction and scatter correction are performed using the CT transmission data.
- A PET/CT examination can include different types of CT scan depending on the CT characteristics, the dose and the use (or not) of oral and/or intravenous contrast agents:

– *Low-dose CT scan*: CT scan that is performed only for attenuation correction (CT-AC) and anatomical correlation of PET findings (with reduced voltage and/or current of the X-ray tube settings), i.e. a low-dose CT is not intended a priori for a dedicated radiological interpretation.
– *Diagnostic CT scan*: CT scan with or without intravenous and/or oral contrast agents, commonly using higher X-ray doses than low-dose scans. Diagnostic CT scan should be performed according to applicable local or national protocols and guidelines.

Common clinical indications

FDG PET/CT is a rapidly evolving imaging modality at both the national and the international levels, with some striking differences between individual countries. FDG PET/CT has become one of the cornerstones of patient management in oncology.

Indications for FDG PET/CT include [10–12, 20, 21], but are not limited to, the following:

- Differentiation of benign from malignant lesions
- Searching for an unknown primary tumour when metastatic disease is discovered as the first manifestation of cancer or when the patient presents with a paraneoplastic syndrome.
- Staging patients with known malignancies.
- Monitoring the effect of therapy on known malignancies.
- Determining whether residual abnormalities detected on physical examination or on other imaging studies following treatment represent tumour or posttreatment fibrosis or necrosis.
- Detecting tumour recurrence, especially in the presence of elevated tumour markers.
- Selection of the region of tumour most likely to yield diagnostic information for biopsy.
- Guiding radiation therapy planning.

Other documents include further indications for FDG PET/CT [10, 20]. The clinical utility of this valuable technology continues to expand in oncology and therefore an exhaustive list of appropriate indications would not be possible or remain final for long.

FDG PET/CT also has an increasingly relevant role in inflammation and infection imaging [23], cardiology and neurology. In these areas the FDG PET/CT procedure may require specific elements not addressed in these guidelines.

Regulatory issues

There is consistent progress in the field, with regular new literature and registration of FDG for several indications by the European Medicines Agency. In the United States, FDG is approved by the Food and Drug Administration for all oncological indications.

Qualifications and responsibilities of personnel

In Europe, the certified nuclear medicine physician who performed the study and signed the report is responsible for the procedure, according to national laws and rules. In the United States, see the SNMMI Guideline for General Imaging [24].

Procedure/specification of the examination

Request

The request for the examination should include sufficient medical information to demonstrate medical necessity and should at least include the diagnosis and questions to be answered.

Review of the medical history

The medical record should be reviewed with a special focus on the diagnosis (type of cancer and known sites), oncological history and relevant comorbidity (especially infection/inflammation and diabetes mellitus). A short interview with the patient and/or family can help clarify some of these issues. Relevant laboratory tests should be considered. The results of prior imaging studies should be available to review, including planar radiography, CT, MRI, bone scanning and FDG PET/CT. Relevant prior studies should be directly compared with current imaging findings when possible. The following list shows all aspects that should be considered in the review:

- Tumour type (if known) and known tumour sites.

- Oncological history and relevant comorbidity (especially infection/inflammation and diabetes mellitus).
- Neurological or psychiatric clinical presentations, including suspected neurological paraneoplastic syndromes.
- Height and body weight (these must be determined precisely in the case of SUV measurements, see below). Weight must be measured directly prior to *each* FDG PET/CT examination (also in the case of longitudinal studies) because body weight often changes during the course of disease.
- Serum glucose, date, time.
- Full overview of current and recently used medication, especially (but not limited to) antidiabetic medication, corticosteroids, growth factors and sedatives. In the case of therapy evaluation: type and date of last therapeutic intervention.
- Results of other imaging tests (especially CT, MRI and previous PET/CT), including dates of acquisition, full reports and, if possible, DICOM data of the referred studies for comparison.
- Other examinations performed earlier on the same day as the PET/CT is scheduled. If intravenous contrast agent has been used or specific preparation followed in the 24 – 48 h prior to the FDG PET/CT examination, the situation should be evaluated and noted; if possible such circumstances should be avoided in patient scheduling.
- Allergy to contrast agents. If an FDG PET/CT examination with intravenous CT contrast agent is strictly necessary the referring physician must indicate the premedication protocol to prepare the patient.
- Renal function. Creatinine and/or glomerular filtration should be evaluated, according to national guidelines, if intravenous contrast agent is to be used. If renal function is suboptimal and an FDG PET/CT examination with intravenous CT contrast agent is necessary, then the referring physician can initiate the protocol for prevention of nephrotoxicity (hydrate the patient and repeat the blood test, and if necessary prescribe medication for prevention of nephrotoxicity).

Patient preparation and precautions

The main purpose of patient preparation to reduce tracer uptake in normal tissue (kidneys, bladder, skeletal muscle, myocardium, brown fat) while maintaining and optimising tracer uptake in the target structures (tumour tissue) and keeping patient radiation exposure levels as low as reasonably possible (ALARA). A generally applicable protocol is outlined below.

Pregnancy (suspected or confirmed)

For any diagnostic procedure in a female patient known or suspected to be pregnant, a clinical decision is necessary in which the benefits are weighed against the possible harm. The International Commission on Radiological Protection (ICRP) reports that for an adult patient the administration of 259 MBq (7 mCi) of FDG results in an absorbed radiation dose of 4.7 mGy to the nongravid uterus (i.e. 1.8×10^{-2} mGy/MBq) [25]. Direct measurements of FDG uptake in a case study suggested somewhat higher doses than are currently provided in standard models [26]. A pregnancy test may help with the decision, provided the 10 day postovulation blackout is understood. In the event of doubt and in the absence of an emergency, the 10 day rule should be adopted. In Europe, national guidelines may apply.

Breastfeeding

The ICRP does not recommend interruption of breastfeeding after FDG administration since little FDG is excreted in the milk [25]. However, as the lactating breast accumulates FDG [27], it is suggested that contact between mother and child be limited for 12 h after injection of FDG to reduce the radiation dose that the infant receives from external exposure to radiation emitted by the mother. It is recommended that the infant be breastfed just before injection, to maximise the time between the injection and the next feed. Breast milk may be expressed and fed to the infant via a bottle for 12 h to help minimise the interruption in close, prolonged contact between the infant and the mother.

Instructions to patients

Nondiabetic patients should not consume any food, simple carbohydrates or liquids other than plain (unflavoured) water for at least 4 h prior to the start of the FDG PET/CT study (i.e. with respect to the time of injection of FDG). In practice, this means that patients scheduled to undergo the FDG PET/CT study in the morning should not eat after midnight and preferably should have only a light meal (no alcohol and only a small amount of carbohydrates) during the evening prior to the FDG PET/CT study. Those scheduled for an afternoon FDG PET/CT study may have a light breakfast at least 4 h prior to the time of their PET/CT examination appointment. Medication can be taken as prescribed.

- Adequate prehydration is important to ensure a sufficiently low concentration of FDG in the urine (fewer artefacts) and for radiation safety reasons. For example, consumption of 1 L of water during the 2 h prior to injection is suggested. Where necessary, account for the volume of water in oral contrast agent if it is to be given for a diagnostic CT scan.

- Coffee or caffeinated beverages are not recommended because even if "sugarless" they may contain traces of simple carbohydrates and have the potential to induce excitant effects; this may also be the case for "sugar-free" beverages.
- Parenteral nutrition and intravenous fluids containing glucose should be discontinued at least 4 h before the time of FDG injection. In addition, the infusion used to administer intravenous prehydration must not contain glucose.
- During the injection of FDG and the subsequent uptake phase, the patient should remain seated or recumbent and silent (this is particularly true for head and neck cancer patients) to minimise FDG uptake in muscles. The patient should be kept warm starting 30 – 60 min before the injection of FDG and continuing throughout the subsequent uptake period and examination to minimise FDG accumulation in brown fat (especially relevant in winter or if the room is air-conditioned).
- Patients must avoid strenuous exercise for at least 6 h before the FDG PET/CT study, and preferably for 24 h.
- Patients should void immediately prior to the PET/CT examination to reduce bladder activity.
- The patient should be able to lie still in the PET/CT system for the duration of the examination (20 – 45 min). A specific inquiry about claustrophobia at the time the patient is scheduled for the study may decrease the number of nondiagnostic studies and cancellations, and allow premedication planning.
- If possible, the patient should put his/her arms above the head; proper support devices (e.g. foam pallets) provided by the manufacturers should be employed whenever feasible.
- When a diagnostic contrast-enhanced CT examination with intravenous contrast agent is to be performed, specific indications must be followed (see later in these guidelines).

Serum glucose level before FDG administration

The main objectives of patient preparation with at least 4 h of fasting are to ensure low blood glucose and low insulinaemia, as insulin is directly responsible for glucose uptake by nontumour cells [28]. Although efforts should be made to decrease blood glucose to normal levels (typically 4 – 7 mmol/L) and insulinaemia to low levels, if the study is indicated in a patient with unstable ("brittle") or poorly controlled diabetes (often associated with infection), hyperglycaemia should not represent an absolute contraindication to the study, as fasting hyperglycaemia does not hamper the clinical value of FDG PET [28]. Therefore we recommend the same advice and suggest recording the blood glucose level and any other information that could be relevant for interpretation of the examination.

Blood glucose level must be measured prior to administering FDG. A glucose meter (or glucometer) or a similar bedside device capable of performing overall blood glucose measurements can be used for this purpose, but a blood glucose test *must* be performed with a calibrated and validated method if plasma glucose level is to be used for correction of SUV measurements [29].

It is good practice to check the blood glucose of the patient on arrival at the imaging centre to ensure the level is not too low (not below 4 mmol/L, about 70 mg/dL) or too high, since this may avoid an unnecessary wait. For diabetic patients, it is suggested that blood glucose level is checked upon arrival in order to initiate, if necessary, manoeuvres to lower the blood glucose level as soon as possible. Certain patients can be asked to arrive at the imaging centre earlier than usual to allow more time to correct possible hyperglycaemic situations.

For clinical studies:

- If the plasma glucose level is lower than 11 mmol/L (about 200 mg/dL), the FDG PET/CT study can be performed.
- If the plasma glucose level is higher than or equal to 11 mmol/L (about 200 mg/dL), the FDG PET/CT study should be rescheduled or the patient excluded depending on the patient's circumstances and the trial being conducted.

For research studies:

- The recommended upper plasma glucose levels may range between 7 and 8.3 mmol/L (126 mg/dL and 150 mg/dL) [30]; the upper threshold should be specified in the study protocol. Patients who fall outside the specified range of serum glucose levels are often excluded from the study, but reference should be made to the specific study protocol in reaching this decision.

It should be stated whether the SUV reported is corrected for glucose and, if so, values should be given with and without glucose correction. Glucose levels should be recorded and reported, to allow the calculation of glucose corrected SUV post hoc. SUV may be reported with glucose correction although this is not common practice in many clinical centres. Note that specifically in response assessment studies, blood glucose levels may change with therapy, and it is strongly recommended that blood glucose levels be measured using validated and calibrated methods (no bedside devices) during sequential FDG PET/CT studies. There are few studies in the literature using glucose normalised SUVs and there is no clear evidence that glucose normalisation improves response monitoring or prediction of outcome as compared to uncorrected SUVs. It is also unclear whether the concept of glucose normalisation is valid for

malignant tumours. Glucose normalisation implies that glucose metabolic rates are tightly regulated. In some malignancies with unregulated glucose metabolic rates, uncorrected SUVs can be more stable than glucose corrected SUVs [31].

Reduction of the blood glucose level by administration of insulin can be considered, but the FDG PET/CT study should also be postponed depending on the type and route of the administration of insulin. Insulin should not be given to reduce glucose levels (this leads to greater muscle uptake of FDG) unless the interval between administration of insulin and administration of FDG is more than 4 h. The preferred route of administration is a subcutaneous injection. If insulin is administered it should be rapid-acting insulin (which reaches the bloodstream 15 min after injection, peaks at 60 min and is effective for 2 – 4 h). Other insulin types that are not recommended for immediate or delayed FDG PET/CT imaging are: regular or short-acting insulin (which reaches the bloodstream 30 min after injection, peaks at 2 – 3 h and is effective for 3 – 6 h), intermediate-acting insulin (effective for 12 – 18 h) or long-acting insulin (effective for 24 h). It is also possible to lower blood glucose in patients just above the cut-off threshold by asking them to hydrate while ambulating and recheck the blood glucose periodically until an acceptable level has been achieved. Recently, intravenous administration of insulin before FDG administration has been discussed, but it has not yet been validated [32].

Diabetes

The following recommendations apply to patients with diabetes mellitus:

Type II diabetes mellitus (controlled by oral medication)

- The FDG PET/CT study should preferably be performed in the late morning.
- Patients must comply with the fasting rules indicated above.
- Patients continue to take oral medication to control their blood sugar. If intravenous contrast agent is going to be administered, metformin should be discontinued at the time of the procedure and withheld for 48 h after the procedure (see below).

Type I diabetes mellitus and insulin-dependent type II diabetes mellitus

- Ideally, an attempt should be made to achieve normal glycaemic values prior to the FDG PET/CT study, in consultation with the patient and his/her attending medical doctor.
- There are three options for scheduling the FDG PET/CT study:

  1. It can be scheduled for late morning or midday. The patient should eat a normal breakfast by early morning (around 7.00 a.m.) and inject the normal amount of insulin. Thereafter the patient should not consume any more food or fluids, apart from the prescribed amount of water. FDG should be injected no sooner than 4 h after subcutaneous injection of rapid-acting insulin or 6 h after subcutaneous injection of short-acting insulin. FDG administration is not recommended on the same day after injection of intermediate-acting and/or long-acting insulin.
  2. It can be scheduled for early morning. The presence of intermediate-acting insulin administered the evening before should not interfere with the PET/CT study and glycaemia will probably still be under control. If long-acting insulin has been used the evening before, there could be a slight interference with the PET/CT study. Thus, if this is the preferred schedule, intermediate-acting (instead of long-acting) insulin is recommended. The patient should eat a normal breakfast after the PET/CT study and inject the normal amount of insulin.
  3. In patients on continuous insulin infusion, if possible the FDG PET/CT study should be scheduled for early in the morning. The insulin pump should be switched off for at least 4 h prior to FDG administration. The patient can have breakfast after the FDG PET/CT study and switch on continuous insulin infusion.

Kidney failure

FDG imaging can be performed in patients with kidney failure, although the image quality may be suboptimal and prone to interpretation pitfalls [33].

Recommendations for image optimisation in specific circumstances, and extra notes

- There is no reason for routine administration of sedatives (e.g. short-acting benzodiazepines) in adult patients. Sedatives may be considered in the case of tumours in the head and neck region to reduce muscle uptake or in claustrophobic patients. A number of agents have been tried and are being tested to reduce brown fat uptake (e.g. 5 mg of intravenous diazepam, administered 10 min prior to FDG [34], or 80 mg of propranolol given orally 2 h before FDG administration [35]), but conflicting results have been reported [36]. Patients should be instructed not to drive a car and to travel home accompanied after sedation.

- When the patient is referred for the evaluation of a lesion in the heart or very close to the myocardium, additional dietary recommendations can be helpful. While there are many options for decreasing normal glucose uptake by the myocardium, common recommendations may include instructions for the patient to follow a low carbohydrate diet for 24 h prior to the PET/CT study, or at least a low carbohydrate meal before starting the 6 h period of fasting before the study [37, 38]. The low carbohydrate diet helps switch the myocardium from using glucose as an energy source to using fatty acids, reducing the uptake of glucose by the myocardium.
- Clinical value may be added to a whole-body or torso FDG PET/CT scan by adding a dedicated brain FDG PET/CT scan, which may be done on the same injected dose and in a single session before the whole-body or torso scan. This can be achieved in a short 5 mm single field of view (FOV) head acquisition following the guidelines for acquisition and reconstruction of brain FDG PET/CT images [39]. The technical qualities of the brain scan as part of a whole-body FDG PET/CT scan are not sufficiently high for detailed diagnostic purposes. This pertains to resolution, voxel dimensions, signal to noise ratio, head fixation issues and possibly reconstruction algorithms. Thus, a dedicated brain FDG PET/CT scan may be added. The indications include primarily neurological or psychiatric clinical presentations, and suspected paraneoplastic disease (including limbic encephalitis). Many of these patients have a negative whole-body or torso FDG PET/CT scan, and the brain FDG PET/CT scan adds value by documenting the existence and extent of functional damage or abnormalities (regional inflammatory or epileptiform activity, defects following inflammation or from other conditions). The brain FDG PET/CT scan may help the differential diagnosis in patients in whom a tumour cannot be identified (neurodegeneration, toxic encephalopathy, primary neuroinfection). Further, this strategy allows simultaneous treatment monitoring of activity in the tumour and paraneoplastic effects in the brain in tumour-positive patients.
- Clinical experience suggests that proper hydration prevents urinary activity from causing problems in image interpretation of abdominal/pelvic tumours. If patients are properly hydrated before imaging, delayed imaging or furosemide intervention is very rarely necessary. It is noted that some centres use transurethral catheterisation in this circumstance but the possible risk of urinary tract infection needs to be carefully weighed against the potential benefits of better image quality. In addition, if the pelvis is a site of particular concern, the CT examination may be performed first from the head to the pelvis followed by the emission acquisition in the opposite direction. This protocol minimises the time delay between the CT and FDG imaging of the pelvis, and thus there is only minimal change in bladder volume between the two scans.
- When a diagnostic CT scan with intravenous contrast agent enhancement is to be performed as part of the FDG PET/CT study, indications, contraindications and restrictions have to be assessed by a qualified physician.
- Medication that interacts with intravenous contrast agent (e.g. metformin for the treatment of diabetes) and relevant medical history (e.g. compromised renal function) should be taken into consideration:

– Renal function should be checked prior to contrast agent administration in all patients considered at risk of contrast agent nephrotoxicity. Routine creatinine testing prior to contrast agent administration is not necessary in all patients; the major indications are age over 60 years, history of preexisting renal disease or impairment (including dialysis, kidney transplant, single kidney, renal cancer and renal surgery), history of diabetes mellitus, history of hypertension requiring medical therapy or use of metformin/metformin-containing drug combinations. Patients who do not have one of the above risk factors do not require a baseline serum creatinine determination before intravenous iodinated contrast agent administration. Estimated glomerular filtration rate is a better predictor of renal dysfunction than creatinine level alone. Patients with a high risk of nephrotoxicity are those with creatinine >13 mmol/L (1.5 mg/dL) and/or glomerular filtration <60 mL/min. If renal function assessment is required, a creatinine level and estimated glomerular filtration rate within the preceding 4 weeks is sufficient in most clinical settings, although it seems prudent to shorten this interval for inpatients and those with a new or heightened risk factor of renal dysfunction [40, 41].

– Metformin is an oral hypoglycaemic agent. If intravenous contrast agent is going to be administered, metformin should be discontinued at the time of the procedure and withheld for 48 h after the procedure. If the risk of nephrotoxicity is high, metformin can be reinstituted only after renal function has been reevaluated and found to be normal. If the risk of nephrotoxicity is low, metformin can be reinstituted without the need for renal function assessment. An alternative glucose-controlling drug should be considered during this time [40–42].

– The risk factors for contrast agent-induced nephropathy must be considered. The more important ones include: preexisting renal insufficiency, diabetes mellitus, dehydration or volume depletion, concurrent nephrotoxic drugs, high dose of contrast agent, age greater than 70 years and cardiovascular disease. Patients with normal renal function are at very low risk of contrast agent-induced nephropathy. Recommendations for preventing contrast agent-induced

nephropathy in patients at risk include: adequate hydration, administration of *N*-acetylcysteine, waiting at least 72 h between studies with contrast agent and use of iso-osmolar contrast agent. Discontinuing diuretics, nonsteroidal antiinflammatory agents and aminoglycosides may also decrease the risk of contrast agent-induced renal failure [40, 41].

– Risk factors for adverse reactions to contrast agent must be assessed. A previous reaction to contrast agent is the most important of all the risk factors. Adverse reactions are classified as either idiosyncratic (anaphylactoid) or nonidiosyncratic. Life-threatening reactions are rare. Premedication reduces the risk of recurrent anaphylaxis, but in patients with a history of a severe reaction, an unenhanced CT examination is preferred [40, 41, 43].
– For CT imaging of the abdomen or pelvis, an intraluminal gastrointestinal contrast agent may be administered to improve visualisation of the gastrointestinal tract on CT (unless it is not necessary for the clinical indication or it is medically contraindicated). Contrast agents must only be used in accordance with the recommendations given in section VII. Nowadays, water or water-based contrast agents are often used as an intraluminal contrast agent that provides improved image quality with reduced artefact [44]. Water can be an effective contrast agent allowing better or equal distention in the bowel and better or equal diagnostic clarity compared with routine barium contrast agent.
– Some patients have difficulties such as claustrophobia, dyspnoea or inability to lie still for the duration of the scan. These patients should be carefully evaluated and an effort made to solve the problem with minimum consequences for the patient and the quality of the scan; sometimes, however, a solution cannot be found even if the study is repeated or rescheduled for another day. Occasionally sedatives can be of help in patients suffering claustrophobia. These issues should be noted in order to avoid potential pitfalls and to facilitate interpretation of suboptimal scans.

Radiopharmaceutical

Product: ^{18}F-fluoro-2-deoxyglucose (FDG)
Nuclide: Fluorine-18
Dosage/activity: Dependent on the system, time per bed position and the patient's weight
Administration: Intravenous
Synthesis and quality control: Conform to the European Pharmacopoeia in Europe or the US Pharmacopeia in the US

Recommendations for FDG dose and administered activity

Recommendations for FDG administered activity

The minimum recommended administered FDG activity and PET acquisition duration for each bed position must be adjusted so that the product of the FDG activity and PET acquisition duration is equal to or greater than the specifications set out below. Therefore, one may decide to apply a higher activity and reduce the duration of the study or, *preferably*, to use a reduced activity and increase the study duration, thereby keeping ALARA principles in mind as well.

In these guidelines two recommendations are provided for determining the minimum FDG administered dose in adults, which assume a linear and a quadratic [45] relationship, respectively, between PET acquisition time per bed position, patient weight and recommended FDG activity. Compared with linear activity prescription, the quadratic scheme results in a slightly higher administered activity for patients >75 kg; this compensates for the lower signal to noise ratio (and hence degraded image quality) due to excessive attenuation, which occurs when linear activity prescription is applied.

The following specifications are given when imaging sites prefer the use of a linear relationship for pragmatic reasons (minimum acceptable administered activity recommendation):

1. For systems that apply a PET bed overlap of ≤30 %, the minimum recommended administered activity is calculated as follows:
   FDG (MBq)=14 (MBq·min·bed^{-1}·kg^{-1}) × patient weight (kg)/emission acquisition duration per bed position (min·bed^{-1}).

2. For systems that apply a PET bed overlap of >30 %, the minimum FDG administered activity is calculated as follows:
   FDG (MBq)=7 (MBq·min·bed^{-1}·kg^{-1}) × patient weight (kg)/emission acquisition duration per bed position (min·bed^{-1}).

Alternative: This alternative includes using a quadratic relationship between recommended administered FDG activity, weight and duration of emission acquisition [45]. In this case use the above equations to determine the administered activity for a 75 kg patient. Next, multiply this activity by the square of the patient weight/75. This will provide the minimum administered activity.

1. For systems that apply a PET bed overlap of ≤30 %, the minimum administered FDG activity is calculated as follows:
   FDG (MBq)=1,050 (MBq·min·bed^{-1}·kg^{-2}) × (patient

weight (kg)/75)^2/emission acquisition duration per bed position (min·bed^{-1}).

2. For systems that apply a PET bed overlap of >30 %, the minimum FDG activity is calculated as follows:

   FDG (MBq)=525 (MBq·min·bed^{-1}·kg^{-2}) × (patient weight (kg)/75)^2/emission acquisition duration per bed position (min·bed^{-1}).

Specific notes and pitfalls to be considered:

- An exploratory further optimisation is presently being evaluated by EARL [46, 47]. This procedure would allow lowering the administered FDG activity for PET/CT systems with higher sensitivity or improved performance using new enhanced technology (e.g. better time-of-flight performance, continuous bed motion or extended axial FOV, i.e. length of bed position). A prerequisite is that imaging sites first obtain EARL accreditation for that system and subsequently follow the instructions provided by the standard operating procedure (SOP) "EARL procedure for assessing PET/CT system specific patient FDG activity preparations for quantitative FDG PET/CT studies" [47].
- A short emission acquisition duration per bed position could also be balanced by a higher administered FDG activity [48, 49].
- For patients weighing more than 90 kg, increasing the emission acquisition time per bed position rather than increasing the administered FDG activity is recommended to improve image quality. Literature suggests that FDG activities higher than 530 MBq for patients above 90 kg should not be applied for L(Y)SO systems [50].
- A maximum administered FDG activity may be imposed by national law. If this is the case, increasing the emission acquisition time should be pursued to keep administered FDG activity within legal limits.
- If the PET acquisition duration for each bed position can be set separately, then the acquisition duration per bed position may be further reduced by up to 50 % for bed positions outside the thorax and abdomen (i.e. at the level of the head, neck and legs) because overall attenuation in these body regions is lower. The FDG activity must still be calculated assuming the acquisition duration per bed position as used for bed positions at the level of the thorax and abdomen. Systems with continuous motion functionality may increase motion speed twofold outside the thoracic and abdominal regions.
- In all cases the administered FDG activity should not result in activities within the FOV that exceed the peak count rate capability of the PET/CT system in use. The emission acquisition duration should then be increased to keep image quality within acceptable limits.

For children and adolescents, administered FDG activity should adhere to the EANM or SNMMI recommendations on paediatric radiopharmaceutical administration [51, 52] or national activity limits, if national limits are lower. Furthermore, there are specific guidelines for FDG PET/CT in paediatric oncology [22].

Materials for preparation and administration of FDG and contrast agent

The following materials and set-up are recommended:

- Weighing scales that are accredited and checked at least annually. Scales should be accurate to within 1 kg.
- Equipment for measuring height, which should be accurate (to within 0.5 cm) and maintained regularly.
- Bedside glucose meter to check serum glucose. Note that many bedside methods do not have sufficient precision to be used for SUV glucose correction [29].
- A three-way valve system for administering FDG and flushing with physiological saline is usually used. However, if automated bedside administration systems are used, then other types of lines may be required to obtain the same flushing and administration results.
- A programmable fluid injector with at least two fluid containers for intravenous administration of contrast agent. Only if a fluid injector is not available may intravenous contrast agent be injected manually, although two-phase contrast agent protocols cannot be carried out.
- First-line emergency drugs and equipment should be in the examination room when a diagnostic CT scan with intravenous contrast agent is to be performed [43]. Emergency devices and drugs are to be available according to national and hospital procedures.
- In the case of manual administration:

– An indwelling intravenous device is used to administer the FDG once the blood glucose has been determined. Make sure that if there is a needle on the syringe it is free of FDG.
– Flush and rinse out the administration syringe with at least 10 mL of normal saline (NaCl 0.9 %) – or solutions without glucose – using the three-way valve.

- In the case of automated administration:

– Make sure that the automated system is able to administer a net FDG activity within 3 % accuracy (this must be ensured by the manufacturer and verified by the user), i.e. the actual administered activity may not deviate by more

than 3 % from that indicated by the device. Follow the instructions provided by the manufacturer.

Procedure for preparation and administration of FDG and contrast agent

- Report any problems with FDG administration and image the injection area if extravasation is suspected.
- The administration system and/or administration lines and intravenous access can be removed after tracer administration (unless CT contrast agent is to be administered subsequently by intravenous injection).
- Residual activity in administration lines and intravenous access should be measured in order to derive net administered FDG activity; procedures and recommendations are detailed in the UPICT oncology FDG-PET CT protocol [30].
- Ambient conditions of the waiting room should help create a stress-free environment and a warm temperature. Give the patient extra blankets if necessary.
- Ask patients to lie or sit as calmly as they can, and not to talk. Provide comfortable beds or chairs. They may go to the toilet while waiting, preferably more than 30 min after injection. Ask patients to use the bathroom to empty their bladder 5 min before the start of the FDG PET/CT study.
- When dedicated brain imaging is indicated, additional preparation is necessary [39]. Patients should be positioned comfortably in a quiet, dimly lit room several minutes before FDG administration and during the uptake phase of FDG (at least 20 min). They should be instructed not to speak, read or be otherwise active. If possible, they should keep their eyes closed during the uptake phase of FDG. It is desirable to have the cannula for intravenous administration in place 10 min before FDG administration.
- Intense bladder or ureter activity can impair the interpretation of lesions in the pelvis and retroperitoneum. Therefore, during the waiting period patients may be asked to drink another 500 mL of water. If a patient is unable to hydrate orally, this amount can be given in the form of normal saline intravenously, provided such a fluid load is not medically contraindicated, e.g. due to impaired renal function or poor cardiac function. Loop diuretics (e.g. intravenous furosemide) can occasionally be given, although this is rarely necessary.
- The recommended interval between FDG administration and the start of acquisition is 60 min. However, for clinical trials this may differ depending on the disease and the aims of the study. Any such variation should be clearly stated in the study protocol. The actual interval should be recorded, i.e. the time between FDG injection and imaging should be reported. Be aware that this is usually not equal to the FDG activity assay or calibration time. Note that consistency of SUV measurements (in-house and when compared to the literature) depends on strict observance of the uptake time, and therefore a 60 min interval is recommended with an acceptable range of 55 – 75 min [30]. When repeating an FDG PET/CT study in the same patient, especially in the context of therapy response assessment, it is essential to apply the same uptake interval to within 10 min [30]. In addition, the use of the same PET/CT system and identical acquisition and reconstruction settings should be applied when making multiple examinations in the same patient.

Protocol/image acquisition

PET acquisition protocol

- Axial anatomical scan coverage: For most oncology indications covering the range from the base of the skull to the mid-thigh is sufficient. A longer scanning trajectory of the whole body may be used if appropriate. Extended whole-body examinations are performed in patients with tumours that show a high probability of metastases in the head, skull, brain and lower extremities. In patients with tumours with a high risk of head and brain metastasis but not metastasis in the lower extremities (e.g. lung cancer), it may be appropriate to perform an extended torso FDG PET/CT scan including the brain in the same scan [53]. Limited-view tumour imaging can be considered for follow-up examinations if the disease is restricted to a defined region (e.g. solitary pulmonary nodule, suspicion of lung cancer, examination of hilar lymph nodes, head and neck tumours, assessment of therapy response). If limited-view imaging is performed in the setting of a longitudinal study, the uptake time of the lesion being studied should be the same (to within 5 min) across all longitudinal imaging studies in the same patient.
- Generally, the patient should be positioned with the arms elevated and supported above the head to avoid beam-hardening artefacts in the abdominal and pelvic regions as well as artefacts caused by truncation of the measured FOV. If the patient is not able to keep the arms elevated above the head, one arm can be kept above the head with the other positioned alongside the body, or both arms can be positioned alongside and close to the body. Either way, every attempt should be made to avoid CT truncation. When using systems with extended CT FOVs, the arms may be positioned alongside the body to enhance patient comfort provided CT truncation is avoided.

- For examination of head and neck tumours a two-step protocol may be helpful [54]:

  1. Head and neck portion with the arms down, then
  2. Apex of the lung through the mid-thigh with the arms up.

- If the FDG PET/CT data are used for radiation planning, the examination should be performed in the position used for radiotherapy treatment, employing the same dedicated positioning devices as are used in the radiotherapy department (e.g. the same radiotherapy table top, laser alignment, immobilisation devices and measures) [15]. These guidelines consider static acquisitions only. Radiotherapy planning may increasingly require respiratory gating, too, but this is not covered in the present guidelines.
- When dedicated brain imaging is indicated [39]:

– Patient preparation should be as for brain scan [39]
– Arms down, head fixed in a head holder
– CT topogram of head, followed by
– Low-dose CT scan, followed by
– Single FOV PET acquisition
– 5 min acquisition 45 – 50 min after injection with injected doses of 300 – 400 MBq. If the suspected underlying indication for the brain scan is probably not cancer-related (dementia, etc.), the emission scan can be acquired earlier, up to 30 min after injection. However, identical time frames must be used for the same indications to render the results comparable.
– Arms down followed by one of the protocols described in the next section (CT protocols for the FDG PET/CT study).
– Brain image reconstruction performed independently for body scan following guidelines.

- In general, FDG PET/CT is performed using a protocol comprising a scanogram/scout scan/topogram and a low-dose CT scan for attenuation correction (CT-AC) and anatomical correlation.
- The CT-AC scan should be performed while the patient continues tidal or shallow breathing. In the case of CT systems with six or fewer rings, a protocol using breath hold in normal expiration should be considered for the duration of scanning the thorax and upper abdomen.
- A standard diagnostic CT scan with intravenous contrast agent may, if appropriate, be performed. Several strategies for performing PET/CT studies that include diagnostic CT imaging are provided in detail below.
- Images should be reviewed before the patient leaves the department to ensure that the examination is technically satisfactory (i.e. that the clinical question can be addressed properly) and to assess any need for additional imaging or urgent contact with the referring physician.
- Online random correction should be based on the 'delayed coincidence time window' technique or random correction using a model based on (block) singles count rates.
- During patient registration with the PET/CT system, users should carefully enter all information into the PET/CT console correctly. This includes, but may not be limited to, patient height and body weight, radiopharmaceutical and the net activity administered. Also the assay activity (i.e. FDG activity) and assay time (i.e. activity calibration time) should be noted and reported. In addition, the time of injection (usually not equal to the assay time or activity calibration time) should be noted and reported. If this information cannot be entered into the PET/CT system, it should be reported in the patient or scan report file.
- Decay correction must be 'on' (see also section PET image reconstruction).

CT protocols for the FDG PET/CT study

- CT imaging within the framework of FDG PET/CT studies typically consists of a topogram and a single or multiple helical CT scans.
- CT acquisition parameters (e.g. tube current, voltage, slice thickness, rotation time and pitch) should be chosen with regard to the objective of the CT examination (e.g. attenuation and scatter correction, colocalisation of radiologically equivalent interpretation). Specific local or national dose limits may apply for these types of CT examinations and should always be adhered to. Overall, CT scan parameters should be chosen such that patient exposure is minimised yet dose is adequate to obtain the necessary diagnostic information.
- Ultra low-dose CT scans are now being introduced on some PET/CT systems, often in combination with iterative reconstruction methods (as discussed below), which may be applied to further reduce CT radiation dose.
- For a diagnostic contrast-enhanced CT scan, standard CT settings as suggested by related guidelines and the supervising radiologist or responsible physician should be employed. Modulation of the tube current is encouraged in the absence of metallic implants (e.g. orthopaedic braces) in the coaxial imaging range to lower patient exposure. Depending on the clinical question, intravenous and/or oral contrast agents may be employed. It might be appropriate to perform a diagnostic CT scan for particular regions of the body, followed by a low-dose CT scan of the rest of the body for CT attenuation correction and colocalisation. In some instances, it might be preferable to begin with a low-dose CT scan of the body and then decide to add a diagnostic CT scan of a particular region,

as the findings on the low-dose CT scan may influence the need for a contrast-enhanced or higher resolution regional view.

- High intravenous or intestinal concentrations of contrast agent may cause artefacts in the reconstructed PET images following CT attenuation correction and thus affect quantification. The impact of intravenous contrast agents on the accuracy of attenuation correction is considered acceptable when CT data are collected in the equilibrium or venous phase (i.e. delayed acquisition) and in some centres a contrast-enhanced CT scan only is performed, although uptake in reference regions such as the mediastinum and liver may be affected.
- Arterial phase CT acquisitions should be avoided. In FDG PET/CT studies without the need for advanced quantification, intravenous contrast agents may be used directly (i.e. the CT scan can also be used for attenuation correction) during the FDG PET/CT study because the impact on visual image quality and interpretation is modest. However, deep inspiration for chest CT acquisition will cause a large degree of misregistration and may introduce unacceptable artefacts if the low-dose CT scan (with normal breathing) is replaced by such a diagnostic deep inspiration CT scan.
- Deep inspiration chest CT scans should not be used for attenuation correction when PET quantification is required or intended. Therefore, for attenuation correction, the PET study ideally should be combined with a low-dose CT scan obtained during tidal or shallow breathing or with a contrast-enhanced CT scan obtained during tidal or shallow breathing (as described below).
- The presence of a positive contrast agent (intravenous or oral) may minimally affect the CT attenuation map and, therefore, affects SUV quantification [55]. If this were the only aspect to be taken into consideration, the ideal would be to prohibit CT contrast agent administration. However, in some clinical situations (depending upon tumour type, tumour behaviour or level of anatomical interest), the benefit of CT contrast agents may outweigh the small errors induced in SUV measurement, which may include increased SUV variability. Each protocol should specify the desired approach for the given study. Most importantly, in the same subject, the same approach should be followed at all subsequent imaging time points. In the case of longitudinal studies in which a diagnostic contrast-enhanced CT scan may not be indicated in all PET/CT examinations, strategies 1, 2a and 2b, as indicated below, should be followed.
- If the FDG PET/CT study is performed with the purpose of quantitatively assessing FDG uptake and a diagnostic CT scan is required, the following PET and CT acquisition sequences or strategies should be followed, as indicated in the UPICT Oncology FDG PET/CT protocol [30] and the FDG PET/CT QIBA profile [56]:

– *Strategy 1*: When CT is used for attenuation correction and localisation only (not intended as a clinically diagnostic CT scan):

  - CT topogram, followed by
  - Low-dose CT scan, followed by
  - PET acquisition

– *Strategy 2*: When a contrast-enhanced diagnostic CT scan is also needed, one of the following options must be used:

Strategy 2a (recommended as it avoids any, albeit possibly minimal, impact of intravenous contrast enhancement on attenuation correction and therefore SUV determination):

  - Follow strategy 1
  - Acquire an additional intravenous contrast-enhanced diagnostic CT scan with breathing instructions, if needed

Strategy 2b:

  - Perform an intravenous contrast-enhanced diagnostic CT scan with breathing instruction if needed
  - Follow strategy 1 with a delay of at least 60 s to allow contrast agent to dilute over the body/blood pool

- In the case of protocols or scanning strategies that can be used in clinical practice, i.e. when there is no need for or intention to perform SUV-based quantification, the diagnostic CT scan with intravenous contrast agent may be used for attenuation correction. If contrast-enhanced CT is used for attenuation correction it may alter SUV quantification (<10 % on average). Therefore, the strategies below are for image interpretation based on visual (uptake) assessment only. FDG PET/CT studies performed with the intention of assessing FDG uptake quantitatively should follow the recommendations given above (strategy 1, 2a or 2b). A deep-inspiration thoracic CT scan with a 20 s delay from the beginning of contrast agent infusion can be included as it will provide additional information. However, this CT scan is used neither for attenuation correction nor for PET/CT image fusion, but rather to assess the lung parenchyma with thinner slices (2.5 mm), which is very useful for comparison with previous and/or future studies, and also allows evaluation of thoracic vessels.

– *Strategy 3* (low-dose CT scan):

  - CT topogram, followed by

- A deep-inspiration thoracic CT scan with a 20 s delay from the beginning of the contrast agent infusion (this CT scan is used neither for attenuation correction nor for PET/CT image fusion), followed by
- A whole-body low-dose CT scan (with shallow or tidal breathing) with a 45 s delay after the thoracic CT scan (equilibrium or venous phase) if the thoracic CT scan was performed or with a 60 s delay after the beginning of contrast agent infusion if the thoracic CT was not performed, followed by
- PET acquisition

– *Strategy 4* (diagnostic CT scan):

- CT topogram, followed by
- A deep-inspiration thoracic CT scan with a 20 s delay from the beginning of contrast agent infusion (this CT scan is used neither for attenuation correction nor for PET/CT image fusion), followed by
- A whole-body diagnostic CT scan (with shallow breathing) with a 45 s delay after the thoracic CT scan (equilibrium or venous phase) if the thoracic CT scan was performed, or with a 60 s delay after the beginning of contrast agent infusion if the thoracic CT scan was not performed, followed by
- PET acquisition

- Other specific protocols can be applied depending on the tumour type and the clinical indication.
- Intravenous contrast agent is ideally administered with a programmable fluid injector at a speed of 2.5 ml/s for a catheter of 20G×1.16″ if located in the elbow. If the catheter is placed in other locations, the diameter of the catheter and/or the speed of infusion and delay may need to be adjusted.
- Oral contrast agents allow better delineation of the gastrointestinal tract. A positive contrast agent (for example diluted barium) as well as a negative or water-based contrast agent (for example water or locust bean gum) can be used [44]. High intraluminal concentrations of barium or iodinated contrast agents can cause an attenuation correction artefact in PET images, resulting in an overestimation of FDG accumulation at those sites. These artefacts can be avoided by using a negative contrast agent. However, administration of water only as a negative intraluminal contrast agent itself is associated with fast reabsorption and can cause increased nonspecific FDG accumulation in the bowel [57]. If quantification of the FDG PET/CT studies is required, the use of diluted positive contrast agents only is recommended. The concentration of diluted positive contrast agents should be low enough to guarantee absence of attenuation correction artefacts, and this should be verified for each combination of PET/CT system, PET/CT image reconstruction software and contrast agent being used.
- It should be ensured that the patient is lying within the CT-AC FOV and in the same position as during the PET acquisition. If the system is equipped with extended FOV capabilities this option should preferably be used to avoid CT truncation.
- Metal implants can cause severe artefacts in the CT image. Metal artefact reduction techniques may be used to minimise these artefacts. When the CT data are used for attenuation correction of the PET data, it should be considered that even when using metal artefact reduction techniques, metal implants will likely result in reduced PET image quality and will prevent proper quantification (at and near the metal implant). FDG uptake should be confirmed by inspecting the PET images without attenuation correction.

Pitfalls

- In most PET/CT systems today, the measured FOV of the CT scanner is smaller than that of the PET scanner. Truncating the CT images causes reconstruction artefacts and thus inaccurate quantification of the PET study. When available, truncation correction algorithms may be applied during image reconstruction (and/or during processing of the CT data used for attenuation correction). As the amount of truncation may vary across studies and subjects, it will be difficult to ensure proper quantification across studies and subjects. It is, therefore, *strongly* recommended that any truncation of the CT images is avoided. If available, the use of extended CT and PET FOVs is recommended. It should be noted that truncation of the CT images may occasionally seriously affect scatter correction scaling as well, and may lead to inaccurate quantitative results.
- Make sure that all clocks (including the dose calibrators – the one at the hospital and the one at the external laboratory dispensing the FDG – and the PET/CT system) are synchronised and that this is regularly checked. Consult the local service engineer when needed. Clocks should be synchronised with the official local time to within 1 min (in the case of studies using ^{18}F).

Image reconstruction

PET image reconstruction

The PET emission data must be corrected for geometrical response and detector efficiency (normalisation), system dead time, random coincidences, scatter and attenuation.

- All the corrections necessary to obtain quantitative image data should be applied during the reconstruction process.

- During image reconstruction, matrix sizes and zoom factors should be chosen such that reconstructed voxel sizes are within 3.0 – 4.0 mm in any direction [30, 56].
- When available, time-of-flight information should be used during reconstruction.
- Resolution modelling during reconstruction or other new reconstruction or image processing methods may be applied. In the case of multicentre studies or when quantification is required, the use of these methods typically entails additional filtering during or after image reconstruction in order to meet the standardised/harmonised quantitative PET/CT system performance specifications, as detailed below. When these multicentre standards cannot be met, these reconstruction methods and settings should not be used for quantification of FDG uptake.
- Spatial filters applied during or after reconstruction should not exceed a full-width at half-maximum of 7 mm, not even to mitigate Gibbs artefacts when using resolution modelling. In these cases the use of reconstructed images generated without resolution modelling should be considered.

It is good clinical practice to perform reconstructions with and without attenuation correction to identify potential reconstruction artefacts caused by the CT-AC. Both attenuation-corrected (AC-PET) and non-attenuation-corrected PET (NAC-PET) images should be available for interpretation and lesions seen on the AC-PET images may need to be checked on the NAC-PET images, particularly when adjacent to highly attenuating materials, such as contrast agent or metal implants.

Further standardisation of reconstruction settings is necessary to obtain standardised and harmonised SUV recoveries. This requires reconstruction settings to be chosen so as to achieve matching convergence and spatial resolution across various systems and sites, especially within a multicentre setting [48, 58, 59]. These reconstruction settings should thus be chosen to meet the multicentre QC harmonising specifications for both calibration QC and image quality/SUV recovery QC, for example as described on the EARL website [46]. Indicative reconstruction settings for each system type are provided on request through the EARL website [46].

It may be appropriate to perform multiple PET reconstructions with different reconstruction settings. For quantitative assessment of the FDG PET/CT study, the EARL-approved reconstruction settings, which meet the standardised performance standards, should be used. An additional reconstruction designed for optimal visual assessment may be performed for qualitative interpretation only. This reconstruction may be performed, for example, in order to maximise lesion detectability or to meet local preferences for visual interpretation of the FDG PET/CT study, as was suggested and demonstrated by Lasnon et al. [60]. Similar strategies may be applied for different PET/CT systems as well, provided quantitative interpretations/analyses are performed using the EARL-approved reconstruction settings.

CT image reconstruction

For diagnostic CT scans, acquisition parameters should be determined according to specific or national radiology society guidelines. The CT data that are acquired during the PET/CT study are usually reconstructed using filtered back projection. Recently introduced iterative reconstruction methods for CT data may be applied, if available on the PET/CT system. Depending on the CT protocol and the clinical case, separate CT reconstructions may be performed for diagnostic purposes and CT-AC. The reconstructions will probably differ in their slice thickness, slice overlap, filter etc. In addition to the reconstruction kernel that modulates the image characteristics within the slices (i.e. spatial resolution, edge enhancement and noise texture), a longitudinal filter in the z-dimension is often used to optimise the resolution in the axial direction and to modify the slice sensitivity profiles. The measured attenuation values (μ) are normalised to the density of water (μ_{water}) in order to assign a device-independent numerical value in the framework of the reconstruction:

$$\text{CT value} = \text{Hounsfield units} = 1{,}000(\mu - \mu_{\text{water}})/\mu_{\text{water}}$$

In modern CT systems the spatial resolution in the z-direction is almost as high as the transaxial resolution and nearly isotropic, allowing high-quality images in the coronal and sagittal views. Additionally, postprocessing such as volume rendering or maximum intensity projections benefit from using the high-quality reconstructed CT data.

Image analysis and interpretation

Image analysis and SUV calculations

FDG PET images should be displayed with and without attenuation correction. On all slices (of the attenuation-corrected data) quantitative information with respect to size and FDG uptake can be retrieved. Images must be evaluated using software that is able to display fused PET and CT data and use an SUV scale. Monitors used for image viewing should be approved for clinical use in radiology and nuclear medicine. Characteristics and settings of the monitor should be in line with published standards (e.g. the Medical Electrical Safety Standards, IEC 60601-1/EN 60601-1; the Medical ECM Standards, IEC 60601-1-2, EN 60601-1-2; or national guidelines). Moreover, viewing conditions (e.g. background light) must be appropriate to ensure adequate image inspection. Image data should be stored on an approved PACS system and in DICOM format; further details and recommendations regarding image data format can be found in the QIBA FDG PET/CT profile [56].

The presence or absence of abnormal FDG accumulation on the PET images, especially focal accumulation, in combination with intensity of uptake and anatomical size should be evaluated. Absence of tracer accumulation in anatomical abnormalities seen on the CT scan or other imaging may be particularly significant. When appropriate, the report should correlate PET/CT findings with those of other diagnostic tests, interpret them in that context and consider them in relation to the clinical data. For response assessment, the images should be viewed over the same dynamic grey scale or colour scale range, i.e. a fixed colour scale; for example, from SUV=0 to SUV=10 using an inverse linear scale.

Both uncorrected and attenuation-corrected images may need to be reviewed to identify artefacts caused by contrast agents, metal implants and/or patient motion. In clinical trials, criteria for visual analysis should be defined a priori within the study protocol.

SUV is increasingly used in clinical studies in addition to visual assessments. SUV is a measurement of the uptake in a tumour normalised on the basis of a distribution volume. Most of the published literature relates to SUV (normalised to body weight) measurements. SUV normalised to lean body mass (LBM) is referred to as SUL [61], and is a recommended quantitative measure of FDG uptake. SUL should preferably be calculated alongside SUV, as follows:

$$\text{SUL} = \text{Act}_{\text{VOI}}\ (\text{kBq/mL})/\text{Act}_{\text{administered}}\ (\text{MBq})/\text{LBM}\ (\text{kg})$$

The following calculation is applied in the case of plasma glucose correction:

$$\text{SUL}_{\text{glu}} = \text{Act}_{\text{VOI}}\ (\text{kBq/mL}) \times \text{Gluc}_{\text{plasma}}\ (\text{mmol/L})/\text{Act}_{\text{administered}}\ (\text{MBq})/\text{LBM}\ (\text{kg}) \times 5.0\ (\text{mmol/L})$$

where Act_{VOI} is the activity concentration measured in the volume of interest (VOI) and $\text{Act}_{\text{administered}}$ is the net administered activity corrected for the physical decay of FDG to the start of acquisition and corrected for the residual activity in the syringe and/or administration lines and system. LBM is calculated according to the formula of Janmahasatian et al. [62]:

$$\text{LBM}^{\text{M}} = 9{,}270 \times \text{weight}/(6{,}680 + 216 \times \text{BMI})$$
$$\text{LBM}^{\text{F}} = 9{,}270 \times \text{weight}/(8{,}780 + 244 \times \text{BMI})$$

where LBM^{M} and LBM^{F} are the LBM for males and females, and BMI is body mass index (weight/height^2), and weight and height are in kilograms and metres, respectively. These formulas are clearly more realistic than the previously used James formulas [63, 64], which fail at weights greater than about 120 kg [65]. Patient height, weight and gender should be reported to allow other SUV normalisations, such as weight and body surface area. In these cases LBM is replaced by body weight and body surface area, respectively, in the SUL equations given above.

The use of SUL is preferred for response assessment studies when large changes in body weight may occur during the course of the treatment. As stated above, it is recommended plasma glucose levels be measured using validated methodology and that SUL be calculated with and without plasma glucose correction in all response monitoring studies. Note that the measured glucose content ($\text{Gluc}_{\text{plasma}}$) is normalised for an overall population average of 5.0 mmol/L so that the SULs with and without correction for glucose content (SUL_{glu} and SUL, respectively) are numerically practically identical (on average) [49].

Physiological FDG distribution and interpretation criteria

Accumulation of FDG can normally be seen in the brain, heart, kidneys and urinary tract at 60 min after injection [66]. The brain has a high uptake of FDG (about 7 % of injected activity). The myocardium in a typical fasting state primarily uses free fatty acids, but after glucose load it uses glucose. In the fasting state, FDG uptake in the myocardium should be low, but this is variable. Unlike glucose, FDG is excreted by the kidneys into the urine and accumulates in the urinary tract. There is some degree of FDG accumulation in muscles that can be increased following exercise and serum insulin. Uptake in the gastrointestinal tract varies from patient to patient and may be increased, for example, in patients taking metformin. Uptake is common in lymphoid tissue in Waldeyer's ring and in the lymphoid tissue of the terminal ileum and caecum. Physiological thymic uptake may be present, especially in children and young adults. Uptake in brown fat may be observed more commonly in young patients and when the ambient temperature is low. No physiological uptake is noted in bone itself (unless free ^{18}F-fluoride is present as a contaminant), but bone marrow uptake can be present to a variable degree in patients receiving growth factors (granulocyte colony-stimulating factor, G-CSF, and granulocyte macrophage CSF, GM-CSF) as well as in patients with marrow proliferation for other reasons such as infection, inflammation or anaemia, and following chemotherapy.

- Due to the high physiological FDG uptake in the brain, FDG PET/CT is of limited value for detection of brain metastases. Consequently, FDG PET/CT is generally not used for the primary detection or exclusion of brain metastases.
- Increased FDG uptake is observed in many neoplastic lesions, granulation tissue (e.g. wound healing), infections and other inflammatory processes. A detailed description of pitfalls and situations that can lead to false-positive (benign processes that can show FDG uptake) or false-negative FDG PET/CT interpretation has been published [67].

- Patterns of FDG uptake, established CT morphological criteria and correlation with patient history, physical examination and other imaging modalities may be helpful for differentiation between malignant and benign lesions.
- SUVs and related quantitative measures, such as metabolic tumour volume (MTV) and total lesion glycolysis (TLG), have gained increasing importance for therapy response monitoring [30, 61, 68] and for prognostic assessment [5, 69–71].
- There is no single lower limit of the intensity of FDG uptake for the detection of abnormal uptake within lesions as it depends on the degree of contrast between the tumour and its immediate surroundings. This contrast is related to several pathophysiological factors, the most significant of which are histology (FDG avidity of the type of tumour), volume of vital tumour cells, movement during static acquisition (e.g. blurred signals in the case of pulmonary foci) and physiological high uptake in adjacent background. Furthermore, the sensitivity of FDG PET/CT may be reduced in diabetic patients with elevated glucose levels [72].
- Although there are no conclusive data on the optimum interval between chemotherapy and FDG PET/CT, an interval of at least 10 days between the last treatment and the FDG PET/CT examination is generally considered adequate for measurement of response [20]. This is because of the balance between any possible effects on tumour metabolism (such as macrophage impairment) and systemic effects (such as bone marrow activation following bone marrow depression, which may or may not be caused by growth factors). If an interval of 10 days is not possible, FDG PET/CT should be delayed as long as possible after the previous chemotherapy administration (i.e. until as close as possible to the next treatment cycle).
- The effects of growth factors (G-CSF and GM-CSF) on FDG biodistribution (due to enhanced bone marrow uptake) generally last for more than 2 weeks after the final administration [20].
- It is assumed that the (side) effects of radiotherapy are longer-lasting; investigation of patients with head and neck carcinoma treated with radiation have shown that radiation-induced inflammation can be seen on the FDG PET/CT images for 2 – 3 months after the end of treatment [73, 74]. Waiting 2 or 3 months following completion of radiation therapy before obtaining a PET/CT scan is clinically appropriate as patients rarely develop clinical problems in the first 3 months after treatment.
- In patients who have undergone surgery, uptake depends on the extent of surgery, the presence of infection/inflammation in the wound, and how long after surgery images are acquired. For example, there are few visible signs of a mediastinoscopy after 10 days but sternotomy signs will remain visible for months. Following surgery, it is recommended to delay the FDG PET/CT for at least 6 weeks due to postsurgical inflammation if the scan is primarily being done to assess the surgical field.
- FDG PET/CT for diagnostic purposes is generally assessed using visual criteria, looking for focally increased uptake that may represent malignancy in the appropriate clinical context. It is unclear how SUV can contribute to patient assessment, partly because of the considerable variability in the methodology used. However, this document and several others propose harmonised quantitative FDG PET/CT imaging procedures in multicentre studies and harmonised quantitative interpretation criteria to assess treatment response [30].

Documentation and reporting

The report is the main mode of communication between the physician interpreting the imaging study and the referring physician, and frequently leads to relevant changes in patient management [75]. The SNMMI has recently published reporting recommendations for oncological FDG PET/CT imaging [76].

Direct communication

Abnormalities of immediate clinical importance should be directly or verbally communicated to the appropriate healthcare provider if a delay in treatment might result in significant morbidity. An example of such an abnormality would be a lesion with a high risk of pathological fracture. Other clinically significant unexpected findings should also be communicated verbally. Reporting of abnormalities requiring urgent attention should be consistent with the policy of the interpreting physician's local organisation.

Written communication

Written documentation of verbal reporting should be made in the medical record, usually as part of the PET/CT report [75, 76].

Contents of the report

Study identification

The report should include the full name of the patient, medical record number and date of birth. The protocol name of the examination should also be included, as well as the date and time of its performance. The electronic medical record usually provides these data, as well as a unique study number.

Clinical information

At a minimum, the clinical history should include age, gender, weight, height, reason for referral and the specific question to be answered. If known, the diagnosis and a brief treatment history should be provided. The results of relevant diagnostic tests and prior imaging findings should be summarised. Information relevant for reimbursement should also be included.

Procedure description

The type and date of comparison studies should be stated. If no comparison studies are available, a statement should be made to that effect.

Blood glucose level before FDG administration should be documented.

Study-specific information should include the radiopharmaceutical, the amount of injected activity in megabecquerels and/or millicuries, the route of administration (intravenous) and the date and time of administration. The anatomical site of administration is optional, but should be recorded. The name, dose and route of administration of regulated nonradioactive drugs and agents should also be stated. The type of PET/CT system should be specified, but specific equipment information is optional.

A description of the procedure should include the time the patient was examined or the time interval between administration of FDG and the start time of the acquisition. The part of the body that was covered should be described from the start to the end point. The position of the patient (supine or prone) and the position of the arms (elevated or by the sides) should be stated if nonstandard.

Description of the CT part of the examination may be limited to a statement that a low-mAs CT was performed for attenuation correction and anatomical registration of the emission images. However, findings should be reported. If the CT examination was optimised for diagnosis, then a more complete description of the CT protocol and anatomical findings should be provided. Dosimetric parameters should be included as required by regulations; here, DICOM structured reports may facilitate the extraction of the relevant dose information. The report should state whether CT with or without CT contrast agent was used for CT attenuation correction.

Routine processing parameters are usually not stated in the report, but any special circumstances requiring additional processing, such as motion correction, should be described.

Description of the findings

It is good practice to provide a structured report with concise concluding statements intended to answer the specific clinical question(s) posed, if possible. Nevertheless, there is great variation in the style of reporting. Recommendations with regard to biopsy, alternative radiological studies and follow-up should also be included in the conclusion as appropriate [77]. The interpretation provided by an imaging physician is the chief manifestation of the physician's expertise. Its content affects patient management and clinical outcomes, and it is also a legal document [76].

- Quality of the FDG PET/CT study, e.g. limited due to motion artefacts, abnormal biodistribution of tracer (FDG accumulation in muscles and/or brown fat), infiltration of tracer at the injection site or hyperglycaemia. CT-related artefacts should also be mentioned such as metallic artefacts and other information on large patient body habitus when the quality of the study is affected.
- Description of the location, the extent and the intensity (SUV and/or SUL) of pathological FDG accumulation related to normal tissue.
- Description of relevant findings on CT and their relationship to pathological FDG accumulation. FDG uptake may be reported as mild, moderate or intense and compared to the background uptake in, for example, the liver parenchyma (mean SUV 2.0 – 3.0, maximum SUV 3.0 – 4.0). However, criteria for visual interpretation need to be defined for each study protocol and may vary according to the type of cancer and for different tumour locations. Some criteria have already been proposed [78, 79]. Incidental FDG PET and CT findings should be included in the report, particularly if they are of clinical relevance.
- The CT part of the FDG PET/CT report must describe all relevant anatomical findings (some of which may be FDG PET-negative).
- *Limitations*: If necessary, confounding factors that might influence the sensitivity or specificity of the FDG PET/CT study may be mentioned such as small lesions (partial volume effect), inflammatory changes, muscle activity, high blood glucose levels at the time of injection, paravascular infiltration of FDG or tissue injections.
- *Clinical context*: In the body of the report it can occasionally be helpful to address the findings of the study with respect to the clinical questions asked. However, this is more commonly done in the report Summary/Impression. Any interpretation or summary in the body of the report should also be repeated in the report Summary/Impression.
- *Complementary information*: Comparison with previous examinations should be part of the FDG PET/CT report. FDG PET/CT studies are more valuable if they are interpreted in the context of other imaging examinations (CT, PET/CT, MRI etc.) and clinical data.
- *Assessment of response to therapy*: If an FDG PET/CT study is performed in the context of the assessment of response to therapy, the extent and the intensity of the

FDG uptake should be documented and compared to prior measurements, if available. Examples of criteria for therapy response with FDG as a metabolic biomarker have been suggested by The European Organisation for Research and Treatment of Cancer [68]. In 2009, Wahl et al. suggested the so-called PERCIST criteria for assessing solid tumour response [61]. Further, a five-point scale has been proposed for assessment of lymphoma therapy response [80]. Reporting of the change in intensity of the FDG uptake using semiquantitative parameters – expressed as absolute or relative change – can be used for specific dedicated clinical questions. At present, relative changes in SUV during therapy represent the most robust parameters. The reliability of the results reported will depend on having comparable patient preparation, injection and scanning protocols, as well as comparable data analysis.

Summary and diagnosis/impression

- Clearly identify the study as normal or abnormal.
- The question asked in the study requisition should be directly addressed.
- If possible, a definite diagnosis should be stated. Whenever possible this should provide a staging assessment (TNM or other) stating whether there are categories of uncertainty. Alternatively, a qualitative estimate of the likelihood of a diagnosis and the differential diagnoses should be given.
- If appropriate, repeat examinations and/or additional examinations should be recommended to clarify or confirm findings.
- For therapy evaluation, serial studies should be compared, using visual and/or semiquantitative assessment as appropriate.
- Document the communication of urgent or emergency findings to referring physicians or their deputy.

Additional notes

The Royal College of Radiologists provides recommendations on reporting that include relevant aspects that should be taken into account [77]:

- *Limitations of staging*: Every diagnostic test has a threshold. The threshold used may vary depending on the implications for treatment (to optimise diagnostic accuracy or according to treatment intent).
- *Indeterminate lesions and management of uncertainty*: Indeterminate lesions should be the point of clinical–radiological discussion and/or multidisciplinary review, if better characterisation of the lesion will further affect patient management. The options for resolving uncertainties are discussion, further investigation, intervention and active monitoring (watch and wait).
- *Multidisciplinary team meetings*: Multidisciplinary team meetings allow a team approach to patient management to take into account and evaluate all aspects of the disease prior to individualised therapy planning. It should be possible to review all relevant examinations in a multidisciplinary meeting, especially when there is discrepancy between clinical and imaging findings or other diagnostic uncertainty. During the meetings, the results of the discussions should be recorded, and discrepancies noted and, if necessary, reported as an addendum to the imaging report. If additional tests are needed, these should be scheduled as soon as possible. The presence of a reporting radiologist or nuclear medicine specialist is essential [72].
- *Imaging the treated patient or follow-up studies*: The format of the report should mirror the one at baseline. Reports of follow-up studies must include clear statements regarding the detection of new disease as this implies metabolic progression of disease. Also, descriptions on the direction of change, indeterminate lesions and mixed responses and findings at variance with one another that may reflect different pathologies should be stated [30].

For further reading see also the SNMMI's reporting guidance for oncological FDG PET/CT imaging [76], the Royal College of Radiologists' recommendations on reporting [77] and the SNMMI's Procedure Standard for General Imaging.

Definitions of volumes of interest

Recommended tumour uptake metrics

- The maximum SUL or SUV (SUL_{max}, SUV_{max}) is required for each lesion as specified in the study protocol and/or as considered clinically relevant for routine clinical studies. The SUL_{max} and SUV_{max} are measures of the single whole voxel in a particular lesion with highest/maximum uptake. The maximum uptake should be defined on original reconstructed PET images, i.e. no additional rebinning, resampling, smoothing or any other manipulation by the user is allowed.
- Use of a 3D peak VOI (providing SUV_{peak} and SUL_{peak}) may be determined (when possible) using a 3D 1.2 cm diameter (and 1.0 mL volume) spherical VOI [61] positioned such that the average value across all positions within the lesion is maximised. Often this coincides with

the location (not value) of SUV_{max} and SUL_{max}, but this may not necessarily be the case in all situations.

- The TLG and MTV are of increasing interest and these parameters or their change may have prognostic and/or predictive value. These parameters should therefore be reported, if available. These metrics requires a 3D delineation or segmentation of the FDG-avid lesions. 3D VOIs based on percentage of SUV_{max} or SUL_{max} thresholds are frequently used [48, 81] and most widely available. It is recommended, when possible, to include the following 3D region isocontour-based VOIs for reporting TLG and MTV [48, 81, 82]:

– 3D isocontour at 41 % of the maximum pixel value (VOI_{41})
– 3D isocontour at 50 % of the maximum pixel value (VOI_{50})

- The MTV represents the volume of the above given VOI. MTV_{41} is derived using VOI_{41} and MTV_{50} is derived using VOI_{50}. TLG is the product of the VOI average SUV or SUL (SUV_{mean}, SUL_{mean}) multiplied by the corresponding MTV. TLG_{41} is derived using VOI_{41} and TLG_{50} using VOI_{50}.
- In a longitudinal setting the quantitative metrics described above should be derived using the same VOI approach for all FDG PET/CT examinations in the same patient. Changes in SUL, TLG and MTV should be evaluated using the same VOI approach for all PET/CT studies in the same patient.

Some considerations with respect to semiautomated percentage threshold-based delineation methods

The isocontour described as VOI_{41} corresponds best with the actual dimensions of the tumour, but only for higher tumour-to-background values and nonheterogeneous tracer uptakes. This VOI method, however, does not always result in useful tumour definitions because of noise, tracer uptake inhomogeneities in tumour and background and sometimes low tumour-to-background ratios. In that case, a VOI based on a higher isocontour value (e.g. VOI_{50}) should be chosen for all subsequent studies in the same patient when studies are performed for tumour response assessment.

When VOIs are generated semiautomatically, it is not always possible to generate a reliable VOI if there is a high background or an area of high uptake (bladder, heart) close to/adjacent to the lesion, or if there is low uptake in the lesion. Therefore, semiautomatically generated VOIs must be checked visually. Moreover, in the event of tracer uptake heterogeneity, these VOIs may only delineate the most metabolically active part of the tumour. If the VOIs are not reliable and/or do not correspond visually with the lesion, only the maximum SUV (normalised to body weight) and SUL and possibly 3D SUV_{peak} and SUL_{peak} should be used for reporting.

Other tumour segmentation methods have been described for MTV assessment and/or tumour delineation for radiotherapy planning in the literature, such as contrast-oriented methods [48, 83–85], gradient-based methods [86], iterative methods [87] and fuzzy clustering/segmentation methods [88]. These, however, are not routinely used and not widely available for determining SUVs, although several methods do outperform the simple percentage threshold-based methods [83].

The authors realise the importance of using more accurate and improved delineation methods than those recommended above and, indeed, the use of more advanced methods is encouraged [89]. In particular when FDG PET/CT-based tumour delineations are used for radiotherapy planning, the delineation methods used and the tumour segmentation obtained should be critically reviewed and supervised. Specific guidelines for the use of FDG PET/CT in radiotherapy have been published elsewhere [14, 15, 90–92]. VOI methods other than those recommended in these guidelines may be used provided that at least the maximum uptake (SUL_{max}), and possibly the SUL_{peak}, are always determined and reported as well.

Liver uptake assessment

As suggested by Wahl et al. [61], assessment of liver SUL or SUV may be a useful quality index of an FDG PET/CT study. Liver measurements may be assessed by placing a spherical VOI of diameter 3 cm in the right upper lobe of the liver, avoiding malignancies and organ boundaries, as indicated also in the QIBA FDG PET/CT profile [61]. Liver SULs or SUVs should be reported along with lesion SUL or SUV data. The study protocol or clinical guidelines should define acceptable levels for liver SUL and required actions when specifications are not met. For clinical studies that are quantitatively assessed, mean liver SULs are expected to be within 1.0 and 2.2 (and mean liver SUVs within 1.3 and 3.0) [93]. Liver SULs outside this range may indicate incorrect FDG administration or technical issues, and the use of quantitative analysis of the study should be reconsidered and the uncertainties discussed in the study report.

Mediastinal uptake assessment

Measurement of the mediastinal blood pool can be very useful for assessing what is considered normal or physiological FDG uptake. In recent years, it has been used for the interim evaluation of response to therapy in lymphoma. It is calculated by drawing several VOIs inside the thoracic aorta and measuring the (mean) uptake inside the vessel, taking care not to include in the VOI the vessel wall, where uptake can be

slightly higher when there is vascular inflammation. Blood pool SUL measurements are expected to be around 1.2 (and blood pool SUVs around 1.6) [61, 93, 94].

Quality control and interinstitution PET/CT system performance harmonisation

PET/CT system quality control

The impacts of various technical, physics-related and biological factors have been described extensively [58]. The use of SUVs in multicentre oncology FDG PET/CT studies requires a standardised interinstitution calibration procedure in order to facilitate the exchangeability of SUVs between institutions. It is also important that all participating institutions employ similar methodologies. In order to ensure the comparability of SUVs, a minimum set of QC procedures must be performed, including:

- Daily QC (both the PET and the CT component of the PET/CT system)
- Calibration QC and cross-calibration of PET/CT system with the institution's own dose calibrator or against a dose calibrator (e.g. that of an FDG provider) which is generally used to determine patient-specific FDG activities
- Image quality and recovery coefficients (IQRC)

Note that these QC measures do not replace any QC measures required by national law or legislation or those recommended by local nuclear medicine societies. A brief summary of PET and PET/CT QC procedures, specifically recommended here to ensure accurate SUV quantification, is given below.

Daily QC

The aim of daily QC is to determine whether the PET/CT system is functioning well, and specifically to check for detector failure and/or electronic drift. Most commercial systems are equipped with an automatic or semiautomatic procedure for performing daily QC. For some systems, the daily QC includes tuning of hardware and/or settings (such as gains). Thus both the procedure and its name differ among systems. In all cases all daily QC measures and/or daily set-up/tuning measurements should be performed according to the manufacturer's specifications. Users should check whether the daily QC meets the specifications. When available, a daily PET/CT study of a cylindrical phantom filled with a ^{68}Ge or another long-lived positron-emitting isotope may be acquired [95]. This test enables assessment and reduction of longitudinal variability due to calibration error and/or PET/CT system sensitivity drifts. Inspection of uniformity and quantitative accuracy of the reconstructed PET image may help identify technical failures that were not detected using the routine daily QC procedures. In addition, when possible, sinogram data should be inspected to check for detector failure.

Calibration QC and cross-calibration of PET/CT systems

The aim of cross-calibration is to determine the correct and direct (cross- or relative) calibration of the PET/CT system with the institution's own dose calibrator or against another one which is used to determine patient-specific FDG activities [96]. If FDG activity is ordered directly from and supplied by an external supplier, cross-calibration of the PET/CT system should be carried out using a calibration sample supplied by that provider. At the time of writing this version of the guidelines, standard calibration sources for both dose calibrators and PET/CT systems are not yet widely available, although some initiatives are being undertaken [97]. Therefore, at present these guidelines recommend a proper cross-calibration between the dose calibrator used for patient administered activity and the PET/CT system as a minimal standard. Calibration QC procedure, SOP and specifications are provided in the UPICT oncology FDG PET/CT protocol [30] and by EARL [46].

Image quality and recovery coefficient harmonisation

Although correct cross-calibration is guaranteed using the QC procedure described above, differences in SUV quantification may still occur between centres as a result of differences in the reconstruction and data analysis methodology [60, 98]. To this end an IQRC QC procedure has been developed:

- To determine/check the correctness of a calibration and quantification using a noncylindrical (calibration) phantom containing a set of high-contrast spherical objects.
- To measure standardised 'activity concentration or SUV recovery coefficients' as a function of sphere (tumour) size.

The main aim of the IQRC QC procedure is to guarantee comparable quantitative PET/CT system performance with respect to SUV recovery and quantification. Details on the IQRC QC procedure, SOP and standardised specifications are provided by EARL [46].

Minimum frequency of PET/CT system QC procedures

Procedure	Frequency
Daily QC (outlined above)	Daily
Cross-calibration	Quarterly and always immediately following software and hardware revisions/upgrades and immediately following new set-ups/normalisations
IQRC	Annually and always following reconstruction/system software adjustments, especially adjustments to the reconstruction and/or data analysis (region of interest) software/hardware, and following relevant system hardware changes

CT quality control (CT-QC)

Several documents and reports on CT quality control (CT-QC) have been published and are listed below for readers' information. An overview of CT-QC is given in, for example, the "Equipment Specifications" and "Quality Control" sections of the American College of Radiology Practice Guideline for the Performance of Computed Tomography of the Extracranial Head and Neck in Adults and Children, the American College of Radiology Practice Guideline for the Performance of Pediatric and Adult Thoracic Computed Tomography (CT), and the American College of Radiology Practice Guideline for the Performance of Computed Tomography (CT) of the Abdomen and Computed Tomography (CT) of the Pelvis and in IPEM report 91. In addition, CT performance monitoring guidelines are given in the American College of Radiology Technical Standard for Medical Physics Performance Monitoring of Computed Tomography (CT) Equipment.

Additional QC measures

- Alignment of PET and CT images on a PET/CT system should be checked according to the procedure and frequency recommended by the manufacturer.
- Set-up and normalisation for both PET and CT systems should be performed according to the procedure and frequency recommended by the manufacturer.
- All devices involved (PET/CT systems, dose calibrators, well counters, clocks, scales) should be maintained according to the manufacturers' recommendations or following national guidelines [99]. This includes preventive and corrective maintenance required to ensure correct and accurate functioning of the devices.
- Calibration should always be performed or correct (cross-)calibration should be verified (by means of QC) after maintenance and software upgrades.
- The accuracy of scales used to measure height and to weigh patients should be checked at installation and after maintenance and/or according to the procedure and frequency recommended by the manufacturer.

Radiation exposure to the patient

- The radiation dose with FDG PET/CT is the combination of the radiation exposure from the radiopharmaceutical (Table 1) and the CT study. The radiation dose of diagnostic CT is a matter of concern, particularly for paediatric examinations. The mean dose for a CT scan depends on applications, protocols and CT systems. Especially in children but also in adults it is important to optimise the radiation exposure with respect to the diagnostic question. Recent advances in technology have allowed the radiation

Table 1 Radiation dosimetry for FDG

	Adult	15 years	10 years	5 years	1 year
Recommended administered activity at nominal weight (MBq) [51]	See section VII	302	189	120	70
Nominal weight (kg)	–	55	32	19	10
Organ receiving highest dose [25]	Bladder	Bladder	Bladder	Bladder	Bladder
Absorbed dose per unit activity at voiding interval (mGy/MBq) [25]	1.3×10^{-1}	1.6×10^{-1}	2.5×10^{-1}	3.4×10^{-1}	4.7×10^{-1}
Voiding interval (h) [25]	3.5	3.5	3.5	3.0	2.0
Effective dose (mSv/MBq) [25]	1.9×10^{-2}	2.4×10^{-2}	3.7×10^{-2}	5.6×10^{-2}	9.5×10^{-2}

doses to be significantly reduced relative to a conventional CT or PET examination.

- The coefficient for effective dose from FDG in adults is 1.9×10^{-2} mSv/MBq according to ICRP publication 106 [25], i.e. about 3.5 mSv for an administered activity of 185 MBq. The radiation exposure related to a CT scan carried out as part of an FDG PET/CT study depends on the intended use of the CT study and may differ from patient to patient: the CT scan can be a very low-dose scan (with lower tube voltage and current) for attenuation correction only, or as a low-dose or intermediate-dose scan for attenuation correction and localisation of PET lesions, or additionally a diagnostic CT scan can be indicated (in most cases with intravenous contrast agent administration and deep inspiration for chest CT) for a full diagnostic CT examination. The effective CT dose ranges from 1 to 20 mSv and may be even higher for a static high-resolution diagnostic CT examination. Given the variety of CT systems and protocols, the radiation exposure for a FDG PET/CT study should be estimated specifically for a given imaging system and protocol. Guidelines provided by European radiological societies should be consulted regarding effective dose from the CT examination.[1]
- The choice of imaging protocol strongly depends on the clinical question and must be considered for every single case. Paediatric studies require special attention. For the optimisation of FDG PET/CT studies, dose reduction techniques should be considered.

Additional considerations

At the time of writing this new version of the FDG PET/CT guidelines for tumour imaging, several international collaborative activities are being undertaken to optimise the use of FDG PET/CT as a quantitative imaging biomarker. During the drafting of the guidelines we took the following (draft) documents into consideration: (1) the QIBA FDG PET/CT profile [56] and (2) the UPICT oncology FDG PET/CT protocol [30]. In both documents presently being drafted and revised, clear definitions and general recommendations for both system performance and image procedures are provided. The EANM guidelines presented here use these general recommendations and attempt to translate them into standardised imaging procedure standards applicable to the earlier published European FDG PET/CT guidelines. It should be noted that both the previous EANM guidelines and this new version specifically aim to obtain harmonised SUV data in a multicentre setting. Moreover, the present new version provides an update of the earlier version and attempts to address some new insights and technological developments.

History of the document

These guidelines are a joint project of the EANM Oncology Committee, the EANM Physics Committee and the SNMMI Committee on Guidelines. These guidelines provide an update of the previously published FDG PET and PET/CT: EANM Procedure Guidelines for Tumour PET Imaging: version 1.0, and the SNMMI Procedure Guidelines for Tumour Imaging with ^{18}F-FDG PET/CT 1.0, and address new technologies and developments. There have been major changes in some sections, but others may have hardly been changed. Indeed, there is similarity with the previous version and certain sections have not been altered. Moreover, consideration is given to compliance with international initiatives, such as those by the Quantitative Imaging Biomarkers Alliance (QIBA) [30, 56]. In addition, the previous and this version of the guidelines are based on the following three documents:

The procedure guidelines for tumour imaging with FDG PET/CT of the SNMMI: "Procedure guideline for tumour imaging with ^{18}F-FDG PET/CT 1.0." [21].

The German guidelines for FDG-PET/CT in oncology of the Deutsche Gesellschaft für Nuklearmedizin: "FDG-PET/CT in der Onkologie" [99].

The Netherlands protocol for standardisation of quantitative whole-body FDG PET/CT: "Applications of F18-FDG-PET in Oncology and Standardisation for Multi-Centre Studies" [49].

An overview of other and previously published guidelines [10, 21, 61, 68, 78, 99–106] or recommendations can be found in the supplement issue of the *Journal of Nuclear Medicine* 2009 [58].

Acknowledgments We thank the authors of the EANM FDG PET/CT guidelines version 1.0 for their valuable contributions, which in part are also incorporated in this new version of the guidelines. We acknowledge the support of Richard P. Baum, Emile FI Comans, Adriaan A. Lammertsma, Markus N. Lonsdale, Paul K. Marsden, Felix M. Mottaghy, Mike O'Doherty, Anne M. Paans, Cornelia Schaefer-Prokop. Moreover, we thank the EANM committees and national delegates for their critical review of the manuscript. Finally, we gratefully acknowledge the extensive support of the EANM Office in Vienna during the development of these guidelines and thank Andrea Bauer, Katharina Leissing, Vera Buhmann, Sabine Ettinger and Terez Sera.

Conflicts of interest Ronald Boellaard has research grants from Philips Healthcare and Philips Research and is a member of the scientific advisory board of EARL. Wim J.G. Oyen has research grants from Siemens and is a member of the scientific advisory board of EARL. Klaus Tatsch is

[1] It should be noted that the entity "effective dose" does not necessarily reflect the radiation risk associated with this nuclear medicine examination. The effective dose values given in these guidelines are used to compare the exposure due to different medical procedures. To assess the risk associated with this procedure, it is mandatory to adjust the radiation-associated risk factors at least according to the gender and age distribution of the institution's patient population.

CEO of EARL. Fred J. Verzijlbergen is a member of the scientific advisory board of EARL. Wolfgang A. Weber is a member of the advisory board of GE Healthcare. Scott Holbrook is an employee of and has stock in Precision Nuclear LLC and Invivo Molecular Imaging LLC. Thomas Beyer has research grants from Siemens. Arturo Chiti has received travel grants from GE Healthcare and is a member of the scientific advisory board of EARL. Bernd J. Krause is a consultant for GE Healthcare, has received a GE Healthcare travel grant and has received a research grant from Bayer.

Open Access This article is distributed under the terms of the Creative Commons Attribution License which permits any use, distribution, and reproduction in any medium, provided the original author(s) and the source are credited.

References

1. Avril NE, Weber WA. Monitoring response to treatment in patients utilizing PET. Radiol Clin North Am. 2005;43(1):189–204.
2. Bastiaannet E, Groen H, Jager PL, et al. The value of FDG-PET in the detection, grading and response to therapy of soft tissue and bone sarcomas; a systematic review and meta-analysis. Cancer Treat Rev. 2004;30(1):83–101.
3. Borst GR, Belderbos JSA, Boellaard R, et al. Standardised FDG uptake: a prognostic factor for inoperable non-small cell lung cancer. Eur J Cancer. 2005;41(11):1533–41.
4. Erdi YE. The use of PET for radiotherapy. Curr Med Imaging Rev. 2007;3(1):3–16.
5. Geus-Oei LF, van der Heijden HF, Corstens FH, Oyen WJ. Predictive and prognostic value of FDG-PET in nonsmall-cell lung cancer: a systematic review. Cancer. 2007;110(8):1654–64.
6. Hoekstra CJ, Stroobants SG, Smit EF, et al. Prognostic relevance of response evaluation using [F-18]-2-fluoro-2-deoxy-D-glucose positron emission tomography in patients with locally advanced non-small-cell lung cancer. J Clin Oncol. 2005;23(33):8362–70.
7. Larson SM, Schwartz LH. 18F-FDG PET as a candidate for “qualified biomarker”: functional assessment of treatment response in oncology. J Nucl Med. 2006;47(6):901–3.
8. Vansteenkiste JF, Stroobants SG. The role of positron emission tomography with 18F-fluoro-2-deoxy-D-glucose in respiratory oncology. Eur Respir J. 2001;17(4):802–20.
9. Weber WA. Use of PET for monitoring cancer therapy and for predicting outcome. J Nucl Med. 2005;46(6):983–95.
10. Fletcher JW, Djulbegovic B, Soares HP, et al. Recommendations on the use of F-18-FDG PET in oncology. J Nucl Med. 2008;49(3): 480–508.
11. Delgado-Bolton RC, Fernández-Pérez C, González-Maté A, Carreras JL. Meta-analysis of the performance of 18F-FDG PET in primary tumor detection in unknown primary tumors. J Nucl Med. 2003;44(8):1301–14.
12. Delgado-Bolton RC, Carreras JL, Pérez-Castejón MJ. A systematic review of the efficacy of F-18-FDG PET in unknown primary tumors. Curr Med Imaging Rev. 2006;2(2):215–25.
13. Jiménez-Requena F, Delgado-Bolton RC, Fernández-Pérez C, et al. Meta-analysis of the performance of (18)F-FDG PET in cutaneous melanoma. Eur J Nucl Med Mol Imaging. 2010;37(2):284–300.
14. Grégoire V, Chiti A. PET in radiotherapy planning: particularly exquisite test or pending and experimental tool? Radiother Oncol. 2010;96(3):275–6.
15. Thorwarth D, Beyer T, Boellaard R, et al. Integration of FDG-PET/CT into external beam radiation therapy planning: technical aspects and recommendations on methodological approaches. Nuklearmedizin. 2012;51(4):140–53.
16. Bailey DL, Barthel H, Beuthin-Baumann B, et al. Combined PET/MR: Where are we now? Summary report of the second international workshop on PET/MR imaging April 8-12, 2013, Tubingen, Germany. Mol Imaging Biol. 2014;16(3):295–310.
17. Bailey DL, Barthel H, Beyer T, et al. Summary report of the first international workshop on PET/MR imaging, March 19–23, 2012, Tubingen, Germany. Mol Imaging Biol. 2013;15(4):361–71.
18. Busemann SE, Plachcinska A, Britten A. Acceptance testing for nuclear medicine instrumentation. Eur J Nucl Med Mol Imaging. 2010;37(3):672–81.
19. Thie JA. Understanding the standardized uptake value, its methods, and implications for usage. J Nucl Med. 2004;45(9):1431–4.
20. Boellaard R, O’Doherty MJ, Weber WA, et al. FDG PET and PET/CT: EANM procedure guidelines for tumour PET imaging: version 1.0. Eur J Nucl Med Mol Imaging. 2010;37(1): 181–200.
21. Delbeke D, Coleman RE, Guiberteau MJ, et al. Procedure guideline for tumor imaging with 18F-FDG PET/CT 1.0. J Nucl Med. 2006;47(5):885–95.
22. Stauss J, Franzius C, Pfluger T, et al. Guidelines for 18F-FDG PET and PET-CT imaging in paediatric oncology. Eur J Nucl Med Mol Imaging. 2008;35(8):1581–8.
23. Jamar F, Buscombe J, Chiti A, et al. EANM/SNMMI guideline for 18F-FDG use in inflammation and infection. J Nucl Med. 2013;54(4):647–58.
24. Society of Nuclear Medicine. The SNM procedure guideline for general imaging 6.0. 2010. http://interactive.snm.org/docs/General_Imaging_Version_6.0.pdf. Accessed 23 Nov 2014.
25. ICRP. Radiation dose to patients from radiopharmaceuticals. Addendum 3 to ICRP Publication 53. ICRP Publication 106. Approved by the Commission in October 2007. Ann ICRP. 2008;38(1-2):1–197.
26. Zanotti-Fregonara P, Jan S, Taieb D, et al. Absorbed 18F-FDG dose to the fetus during early pregnancy. J Nucl Med. 2010;51(5):803–5.
27. Hicks RJ, Binns D, Stabin MG. Pattern of uptake and excretion of (18)F-FDG in the lactating breast. J Nucl Med. 2001;42(8):1238–42.
28. Belohlavek O, Jaruskova M. [18F]FDG-PET scan in patients with fasting hyperglycaemia. Q J Nucl Med Mol Imaging. 2014 (in press)
29. Dai KS, Tai DY, Ho P, et al. Accuracy of the EasyTouch blood glucose self-monitoring system: a study of 516 cases. Clin Chim Acta. 2004;349(1–2):135–41.
30. Quantitative Imaging Biomarkers Alliance. Quantitative FDG-PET Technical Committee. UPICT oncology FDG-PET CT protocol. http://qibawiki.rsna.org/index.php?title=FDG-PET_tech_ctte. Accessed 23 Nov 2014
31. Huang SC. Anatomy of SUV. Standardized uptake value. Nucl Med Biol. 2000;27(7):643–6.
32. Caobelli F, Pizzocaro C, Paghera B, Guerra UP. Proposal for an optimized protocol for intravenous administration of insulin in diabetic patients undergoing (18)F-FDG PET/CT. Nucl Med Commun. 2013;34(3):271–5.
33. Minamimoto R, Takahashi N, Inoue T. FDG-PET of patients with suspected renal failure: standardized uptake values in normal tissues. Ann Nucl Med. 2007;21(4):217–22.
34. Rakheja R, Ciarallo A, Alabed YZ, Hickeson M. Intravenous administration of diazepam significantly reduces brown fat activity on 18F-FDG PET/CT. Am J Nucl Med Mol Imaging. 2011;1(1):29–35.
35. Soderlund V, Larsson SA, Jacobsson H. Reduction of FDG uptake in brown adipose tissue in clinical patients by a single dose of propranolol. Eur J Nucl Med Mol Imaging. 2007;34(7):1018–22.

36. Sturkenboom MG, Hoekstra OS, Postema EJ, Zijlstra JM, Berkhof J, Franssen EJ. A randomised controlled trial assessing the effect of oral diazepam on 18F-FDG uptake in the neck and upper chest region. Mol Imaging Biol. 2009;11(5):364–8.
37. Coulden R, Chung P, Sonnex E, Ibrahim Q, Maguire C, Abele J. Suppression of myocardial 18F-FDG uptake with a preparatory "Atkins-style" low-carbohydrate diet. Eur Radiol. 2012;22(10):2221–8.
38. Lum DP, Wandell S, Ko J, Coel MN. Reduction of myocardial 2-deoxy-2-[18F]fluoro-D-glucose uptake artifacts in positron emission tomography using dietary carbohydrate restriction. Mol Imaging Biol. 2002;4(3):232–7.
39. Varrone A, Asenbaum S, Vander BT, et al. EANM procedure guidelines for PET brain imaging using [18F]FDG, version 2. Eur J Nucl Med Mol Imaging. 2009;36(12):2103–10.
40. Bui KL, Horner JD, Herts BR, Einstein DM. Intravenous iodinated contrast agents: risks and problematic situations. Cleve Clin J Med. 2007;74(5):361–4, 367.
41. ACR Committee on Drugs and Contrast Media. ACR manual on contrast media, version 9. ACR, American College of Radiology; 2013. ISBN: 978-1-55903-012-0. http://www.acr.org/quality-safety/resources/~/media/37D84428BF1D4E1B9A3A2918DA9E27A3.pdf. Accessed 23 Nov 2014
42. University of California San Francisco. Department of Radiology and Biomedical Imaging. Contrast administration in patients receiving metformin. http://www.radiology.ucsf.edu/patient-care/patient-safety/contrast/iodinated/metaformin. Accessed 23 Nov 2014
43. European Society of Urogenital Radiology. ESUR guidelines on contrast media. http://www.esur.org/guidelines. Accessed 23 Nov 2014
44. Antoch G, Kuehl H, Kanja J, et al. Dual-modality PET/CT scanning with negative oral contrast agent to avoid artifacts: introduction and evaluation. Radiol. 2004;230(3):879–85.
45. de Groot EH, Post N, Boellaard R, Wagenaar NR, Willemsen AT, van Dalen JA. Optimized dose regimen for whole-body FDG-PET imaging. EJNMMI Res. 2013;3(1):63.
46. EANM Research Ltd (EARL). New EANM FDG PET/CT accreditation specifications for SUV recovery coefficients. http://earl.eanm.org/cms/website.php?id=/en/projects/fdg_pet_ct_accreditation/accreditation_specifications.htm. Accessed 23 Nov 2014
47. Boellaard R, Willemsen AT, Arends B, Visser EP. EARL procedure for assessing PET/CT system specific patient FDG activity preparations for quantitative FDG PET/CT studies. http://earl.eanm.org/html/img/pool/EARL-procedure-for-optimizing-FDG-activity-for-quantitative-FDG-PET-studies_version_1_1.pdf. Accessed 23 Nov 2014.
48. Boellaard R, Krak NC, Hoekstra OS, Lammertsma AA. Effects of noise, image resolution, and ROI definition on the accuracy of standard uptake values: a simulation study. J Nucl Med. 2004;45(9):1519–27.
49. Boellaard R, Oyen WJ, Hoekstra CJ, et al. The Netherlands protocol for standardisation and quantification of FDG whole body PET studies in multi-centre trials. Eur J Nucl Med Mol Imaging. 2008;35(12):2320–33.
50. Masuda Y, Kondo C, Matsuo Y, Uetani M, Kusakabe K. Comparison of imaging protocols for 18F-FDG PET/CT in overweight patients: optimizing scan duration versus administered dose. J Nucl Med. 2009;50(6):844–8.
51. Lassmann M, Treves ST. Paediatric radiopharmaceutical administration: harmonization of the 2007 EANM paediatric dosage card (version 1.5.2008) and the 2010 North American consensus guidelines. Eur J Nucl Med Mol Imaging. 2014;41(5):1036–41.
52. Treves ST, Lassmann M. International guidelines for pediatric radiopharmaceutical administered activities. J Nucl Med. 2014;55(6):869–70.
53. Osman MM, Chaar BT, Muzaffar R, et al. 18F-FDG PET/CT of patients with cancer: comparison of whole-body and limited whole-body technique. AJR Am J Roentgenol. 2010;195(6):1397–403.
54. Beyer T, Antoch G, Muller S, et al. Acquisition protocol considerations for combined PET/CT imaging. J Nucl Med. 2004;45 Suppl 1:25S–35.
55. Mawlawi O, Erasmus JJ, Munden RF, et al. Quantifying the effect of IV contrast media on integrated PET/CT: clinical evaluation. AJR Am J Roentgenol. 2006;186(2):308–19.
56. Quantitative Imaging Biomarkers Alliance. FDG-PET/CT Technical Committee. FDG-PET/CT as an imaging biomarker measuring response to cancer therapy, version 1.05, Publicly Reviewed Version. Quantitative Imaging Biomarkers Alliance; 2013. http://www.rsna.org/uploadedfiles/rsna/content/science_and_education/qiba/qiba_fdg-pet_profile_v105_publicly_reviewed_version_final_11dec2013.pdf. Acessed 23 Nov 2014.
57. Otsuka H, Graham MM, Kubo A, Nishitani H. The effect of oral contrast on large bowel activity in FDG-PET/CT. Ann Nucl Med. 2005;19(2):101–8.
58. Boellaard R. Standards for PET image acquisition and quantitative data analysis. J Nucl Med. 2009;50 Suppl 1:11S–20.
59. Westerterp M, Pruim J, Oyen W, et al. Quantification of FDG PET studies using standardised uptake values in multi-centre trials: effects of image reconstruction, resolution and ROI definition parameters. Eur J Nucl Med Mol Imaging. 2007;34(3):392–404.
60. Lasnon C, Desmonts C, Quak E, et al. Harmonizing SUVs in multicentre trials when using different generation PET systems: prospective validation in non-small cell lung cancer patients. Eur J Nucl Med Mol Imaging. 2013;40(7):985–96.
61. Wahl RL, Jacene H, Kasamon Y, Lodge MA. From RECIST to PERCIST: evolving considerations for PET response criteria in solid tumors. J Nucl Med. 2009;50 Suppl 1:122S–50.
62. Janmahasatian S, Duffull SB, Ash S, Ward LC, Byrne NM, Green B. Quantification of lean bodyweight. Clin Pharmacokinet. 2005;44(10):1051–65.
63. Hallynck TH, Soep HH, Thomis JA, Boelaert J, Daneels R, Dettli L. Should clearance be normalised to body surface or to lean body mass? Br J Clin Pharmacol. 1981;11(5):523–6.
64. James W. Research on obesity. London: Her Majesty's Stationery Office; 1976.
65. Tahari AK, Chien D, Azadi JR, Wahl RL. Optimum lean body formulation for correction of standardized uptake value in PET imaging. J Nucl Med. 2014;55(9):1481–4.
66. Cook GJ, Fogelman I, Maisey MN. Normal physiological and benign pathological variants of 18-fluoro-2-deoxyglucose positron-emission tomography scanning: potential for error in interpretation. Semin Nucl Med. 1996;26(4):308–14.
67. ACR Guidelines and Standards Committee. ACR-SPR practice parameter for performing FDG-PET/CT in oncology. American College of Radiology; 2014. http://www.acr.org/~/media/71B746780F934F6D8A1BA5CCA5167EDB.pdf. Accessed 23 Nov 2014.
68. Young H, Baum R, Cremerius U, et al. Measurement of clinical and subclinical tumour response using [18F]-fluorodeoxyglucose and positron emission tomography: review and 1999 EORTC recommendations. European Organization for Research and Treatment of Cancer (EORTC) PET Study Group. Eur J Cancer. 1999;35(13):1773–82.
69. Itti E, Meignan M, Berriolo-Riedinger A, et al. An international confirmatory study of the prognostic value of early PET/CT in diffuse large B-cell lymphoma: comparison between Deauville criteria and DeltaSUVmax. Eur J Nucl Med Mol Imaging. 2013;40(9):1312–20.
70. Chung HH, Kwon HW, Kang KW, et al. Prognostic value of preoperative metabolic tumor volume and total lesion glycolysis

in patients with epithelial ovarian cancer. Ann Surg Oncol. 2012;19(6):1966–72.
71. Zhang H, Wroblewski K, Liao S, et al. Prognostic value of metabolic tumor burden from (18)F-FDG PET in surgical patients with non-small-cell lung cancer. Acad Radiol. 2013;20(1):32–40.
72. The Royal College of Radiologists. Standards for radiology discrepancy meetings. London: The Royal College of Radiologists; 2007. http://www.rcr.ac.uk/docs/radiology/pdf/Stand_radiol_discrepancy.pdf. Accessed 23 Nov 2014.
73. Andrade RS, Heron DE, Degirmenci B, et al. Posttreatment assessment of response using FDG-PET/CT for patients treated with definitive radiation therapy for head and neck cancers. Int J Radiat Oncol Biol Phys. 2006;65(5):1315–22.
74. Kawabe J, Higashiyama S, Yoshida A, Kotani K, Shiomi S. The role of FDG PET-CT in the therapeutic evaluation for HNSCC patients. Jpn J Radiol. 2012;30(6):463–70.
75. Coleman RE, Hillner BE, Shields AF, et al. PET and PET/CT reports: observations from the National Oncologic PET Registry. J Nucl Med. 2010;51(1):158–63.
76. Niederkohr RD, Greenspan BS, Prior JO, et al. Reporting guidance for oncologic 18F-FDG PET/CT imaging. J Nucl Med. 2013;54(5): 756–61.
77. Padhani AR. Imaging in the evaluation of cancer. In: Nicholson T, editor. Recommendations for cross-sectional imaging in cancer management. 2nd ed. London: The Royal College of Radiologists; 2014.
78. Juweid ME, Stroobants S, Hoekstra OS, et al. Use of positron emission tomography for response assessment of lymphoma: consensus of the Imaging Subcommittee of International Harmonization Project in Lymphoma. J Clin Oncol. 2007;25(5): 571–8.
79. Meignan M, Gallamini A, Haioun C. Report on the First International Workshop on Interim-PET-Scan in Lymphoma. Leuk Lymphoma. 2009;50(8):1257–60.
80. Barrington SF, Mikhaeel NG, Kostakoglu L, et al. Role of imaging in the staging and response assessment of lymphoma: consensus of the International Conference on Malignant Lymphomas Imaging Working Group. J Clin Oncol. 2014. doi:10.1200/JCO.2013.53.5229
81. Krak NC, Boellaard R, Hoekstra OS, Twisk JWR, Hoekstra CJ, Lammertsma AA. Effects of ROI definition and reconstruction method on quantitative outcome and applicability in a response monitoring trial. Eur J Nucl Med Mol Imaging. 2005;32(3):294–301.
82. Frings V, de Langen AJ, Smit EF, et al. Repeatability of metabolically active volume measurements with 18F-FDG and 18F-FLT PET in non-small cell lung cancer. J Nucl Med. 2010;51(12): 1870–7.
83. Frings V, van Velden FH, Velasquez LM, et al. Repeatability of metabolically active tumor volume measurements with FDG PET/CT in advanced gastrointestinal malignancies: a multicenter study. Radiology. 2014;273(2):539–48.
84. Schaefer A, Nestle U, Kremp S, et al. Multi-centre calibration of an adaptive thresholding method for PET-based delineation of tumour volumes in radiotherapy planning of lung cancer. Nuklearmedizin. 2012;51(3):101–10.
85. Schaefer A, Kremp S, Hellwig D, Rube C, Kirsch CM, Nestle U. A contrast-oriented algorithm for FDG-PET-based delineation of tumour volumes for the radiotherapy of lung cancer: derivation from phantom measurements and validation in patient data. Eur J Nucl Med Mol Imaging. 2008;35(11):1989–99.
86. Geets X, Lee JA, Bol A, Lonneux M, Gregoire V. A gradient-based method for segmenting FDG-PET images: methodology and validation. Eur J Nucl Med Mol Imaging. 2007;34(9):1427–38.
87. van Dalen JA, Hoffmann AL, Dicken V, et al. A novel iterative method for lesion delineation and volumetric quantification with FDG PET. Nucl Med Commun. 2007;28(6): 485–93.
88. Hatt M, Lamare F, Boussion N, et al. Fuzzy hidden Markov chains segmentation for volume determination and quantitation in PET. Phys Med Biol. 2007;52(12):3467–91.
89. Cheebsumon P, Yaqub M, van Velden FH, Hoekstra OS, Lammertsma AA, Boellaard R. Impact of [18F]FDG PET imaging parameters on automatic tumour delineation: need for improved tumour delineation methodology. Eur J Nucl Med Mol Imaging. 2011;38(12):2136–44.
90. Chiti A, Kirienko M, Grégoire V. Clinical use of PET-CT data for radiotherapy planning: what are we looking for? Radiother Oncol. 2010;96(3):277–9.
91. Grégoire V, Chiti A. Molecular imaging in radiotherapy planning for head and neck tumors. J Nucl Med. 2011;52(3):331–4.
92. Sattler B, Lee JA, Lonsdale M, Coche E. PET/CT (and CT) instrumentation, image reconstruction and data transfer for radiotherapy planning. Radiother Oncol. 2010;96(3):288–97.
93. Boktor RR, Walker G, Stacey R, Gledhill S, Pitman AG. Reference range for intrapatient variability in blood-pool and liver SUV for 18F-FDG PET. J Nucl Med. 2013;54(5):677–82.
94. Meignan M, Barrington S, Itti E, Gallamini A, Haioun C, Polliack A. Report on the 4th International Workshop on Positron Emission Tomography in Lymphoma held in Menton, France, 3-5 October 2012. Leuk Lymphoma. 2014;55(1):31–7.
95. Lockhart CM, MacDonald LR, Alessio AM, McDougald WA, Doot RK, Kinahan PE. Quantifying and reducing the effect of calibration error on variability of PET/CT standardized uptake value measurements. J Nucl Med. 2011;52(2):218–24.
96. Greuter HN, Boellaard R, van Lingen A, Franssen EJ, Lammertsma AA. Measurement of 18F-FDG concentrations in blood samples: comparison of direct calibration and standard solution methods. J Nucl Med Technol. 2003;31(4):206–9.
97. Gadd R, Baker M, Nijran KS, et al. Protocol for establishing and maintaining the calibration of medical radionuclide calibrators and their quality control. http://www.npl.co.uk/publications/protocol-for-establishing-and-maintaining-the-calibration-of-medical-radionuclide-calibrators-and-their-quality-control. Accessed Sep 2014.
98. Lasnon C, Hicks RJ, Beauregard JM, et al. Impact of point spread function reconstruction on thoracic lymph node staging with 18F-FDG PET/CT in non-small cell lung cancer. Clin Nucl Med. 2012;37(10):971–6.
99. Krause BJ, Beyer T, Bockisch A, et al. FDG-PET/CT in oncology. German guideline. Nuklearmedizin. 2007;46(6):291–301.
100. Bourguet P, Blanc-Vincent MP, Boneu A, et al. Summary of the standards, options and recommendations for the use of positron emission tomography with 2-[18F]fluoro-2-deoxy-D-glucose (FDP-PET scanning) in oncology (2002). Br J Cancer. 2003;89 Suppl 1:S84–91.
101. Coleman RE, Delbeke D, Guiberteau MJ, et al. Concurrent PET/CT with an integrated imaging system: intersociety dialogue from the joint working group of the American College of Radiology, the Society of Nuclear Medicine, and the Society of Computed Body Tomography and Magnetic Resonance. J Nucl Med. 2005;46(7): 1225–39.
102. Lammertsma AA, Hoekstra CJ, Giaccone G, Hoekstra OS. How should we analyse FDG PET studies for monitoring tumour response? Eur J Nucl Med Mol Imaging. 2006;33 Suppl 1:16–21.
103. Miller JC, Fischman AJ, Aquino SL, Blake MA, Thrall JH, Lee SI. FDG-PET CT for tumor imaging. J Am Coll Radiol. 2007;4(4): 256–9.
104. Schelbert HR, Hoh CK, Royal HD, et al. Procedure guideline for tumor imaging using fluorine-18-FDG. Society of Nuclear Medicine. J Nucl Med. 1998;39(7):1302–5.

105. Shankar LK, Hoffman JM, Bacharach S, et al. Consensus recommendations for the use of 18F-FDG PET as an indicator of therapeutic response in patients in National Cancer Institute Trials. J Nucl Med. 2006;47(6):1059–66.

106. Zijlstra JM, Comans EF, van Lingen A, et al. FDG PET in lymphoma: the need for standardization of interpretation. An observer variation study. Nucl Med Commun. 2007;28(10):798–803.
