# Supplementary material for: [18F]FDG PET/CT detection and therapeutic response assessment of primary multisystem extranodal diffuse large B-cell lymphoma without lymph node involvement: a case report
Source: Front Oncol. 2025 Dec 15;15:1697944. doi: 10.3389/fonc.2025.1697944 (PMC12745402; doi:10.3389/fonc.2025.1697944)
Supplement: Supplementary file 2 [file DataSheet2.pdf]

核医学 ^{18}F-FDG PET/CT 报告书写规范

王茜 [1]，李囡 [2]，霍力 [3]，杨吉刚 [4]，艾林 [5]，李河北 [1]，富丽萍 [6]，张建华 [7]，杨志 [2*]

1.北京大学人民医院核医学科，北京 100044；2.北京大学肿瘤医院暨北京市肿瘤防治研究所核医学科，恶性肿瘤发病机制及转化研究教育部重点实验室，北京 100142；3.中国医学科学院 北京协和医学院 北京协和医院核医学科，北京 100730；4.首都医科大学附属北京友谊医院核医学科，北京 100050；5.首都医科大学附属天坛医院核医学科，北京 100070；6.北京中日友好医院核医学科，北京 100029；7.北京大学第一医院核医学科，北京 100034； *通讯作者 杨志 pekyz@163.com

【关键词】核医学；操作规范；正电子发射断层显像/计算机体层摄影术；正电子发射断层显像术；体层摄影术，X 线计算机

【中图分类号】R714.5；R445.1 【DOI】10.3969/j.issn.1005-5185.2021.01.001

随着正电子发射计算机断层显像/计算机断层显像（positron emission tomography/computed tomography，PET/CT）临床应用的增加，氟-18 标记氟代脱氧葡萄糖（flurodeoxyglucose，^{18}F-FDG）PET/CT 的诊断作用得到了临床的广泛认可。^{18}F-FDG PET/CT 报告是核医学医师与临床医师之间的主要沟通方式，报告的内容可以体现影像医师在医疗过程中的作用，作为制订诊疗方案的依据，并作为提供医疗服务的法定证据，用于证明医疗的必要性。因此，重视报告的质量非常必要。由于这种融合成像模式的复杂性，书写一份高质量的 ^{18}F-FDG PET/CT 报告具有一定的挑战性，需要影像诊断医师同时具备多种影像技术能力和一定的相关临床知识。

近年来，美国核医学与分子影像学学会（Society of Nuclear Medicine and Molecular Imaging，SNMMI）、欧洲核医学协会（European Association of Nuclear Medicine，EANM）以及中华医学会核医学分会分别发表了有关 ^{18}F-FDG PET/CT 用于肿瘤显像或感染与炎症显像的相关指导性文件，不同程度地阐述了对报告书写的要求[1-4]。然而，目前国内尚缺乏专门针对报告质量控制的参考标准。为了进一步提升 ^{18}F-FDG PET/CT 对临床诊疗工作的贡献，规范各医疗单位 PET/CT 报告书写内容，北京市核医学质量控制和改进中心组织来自北京市多家大型医院的专家，针对历年来核医学质量控制检查中所发现的问题，经过论证研讨，撰写了此《核医学 ^{18}F-FDG PET/CT 报告书写规范》，对报告所含要素、各要素书写内容及报告审核等方面提出具体的要求，供核医学医师实际工作参考。

1 报告基本要素

完整的 ^{18}F-FDG PET/CT 报告应包括受检者的基本信息、临床病史及检查目的、检查技术及操作过程、检查所见、检查意见等内容（表 1）。书写时应注意行文简洁、条理清晰、用词规范、关键数据完整。

表 1 ^{18}F-FDG PET/CT 报告的基本要素

要素	描述
基本信息	患者姓名、性别、年龄、身高、体重、病历号、送检科室，以及 PET/CT 检查号、检查项目、检查日期、设备型号等
病史及检查目的	患者主诉及简要诊疗经过<br>患者病变类型及部位（如有）<br>本次检查目的
检查技术/程序	核素药物名称、注射剂量、给药途径<br>注射至显像间隔时间<br>血糖水平<br>相关药物使用情况和特殊体位（如有）<br>扫描部位<br>检查程序、对比剂使用情况等
影像所见	异常 ^{18}F-FDG 摄取病灶的位置、大小和程度等<br>PET 异常区域的 CT 或其他临床影像所见（如有）<br>偶然 PET 及 CT 所见<br>其他相关影像检查有阳性发现时与之相对应的 PET 显像结果（如果适用）
影像诊断意见	尽最大可能给出明确结论（有异常/未见异常）<br>解释异常影像所见（不应仅重复异常所见描述），提出鉴别诊断，如可能则提出进一步诊断或随访方案<br>紧急的异常所见应立刻联系相关临床医师或患方联系人

(C)1994-2023 China Academic Journal Electronic Publishing House. All rights reserved. http://www.cnki.net

2　报告中的各要素说明

2.1　基本信息　报告的基本信息应包括患者姓名、性别、年龄、身高、体重、病历号、送检科室，以及PET/CT检查的检查号、检查项目、检查日期、设备型号等。患者基本信息用以保证PET/CT报告的唯一性，便于患者复查时个人多次检查之间的对比，也有利于报告归档、存储、后期在随访及特殊查询时调用。建议使用电子病历系统的单位将上述信息尽可能通过信息化手段直接生成，减少人工二次操作错误，便于溯源。

2.2　病史及检查目的　对病史的描述应包括：疾病的诊断时间或主要症状表现及出现时间（未明确诊断情况下）、相关实验室/影像学/病理学检查结果、主要治疗过程以及可能对影像结果产生影响的药物使用情况及既往手术史等。上述信息可通过询问患者及家属、临床主管医师或查阅在线病历的方式获取。检查目的代表患者的适应证及检查需回答的主要临床问题。PET/CT检查前了解患者的临床病史和检查目的可提示检查的必要性，也有助于核医学医师提供准确、恰当的PET/CT报告。

2.3　检查技术与程序　由于设备、检查方案、患者自身条件等诸多因素会对PET/CT图像质量、标准摄取值（standardized uptake value，SUV）测量值甚至影像判读结果产生影响，故报告中应对相应的检查技术及操作过程做如实的记录，这不仅可作为影像判读和后续附加检查的参考，还可作为影像质量的判断依据。记录内容应包括患者血糖水平、显像剂名称、注射活度、给药时间及给药途径、图像采集时间、辅助干预措施（如水化情况、利尿剂、镇静剂、胰岛素使用等）及扫描参数（采集模式、床速、床位数量、扫描范围）。额外增加的PET/CT显像方案（如延迟显像）亦应详细记录采集时间、范围、扫描速度等可能影响SUV值测量的显像条件；如在PET/CT检查中使用诊断CT及对比剂应记录。

2.4　影像所见　由于PET/CT为大视野成像，所获得的图像包括PET、CT及两者的融合图像，图像数据信息量大，为了避免病变遗漏，报告时建议按照采集范围从上至下或按照系统性病变的观察顺序对异常所见进行描述，并提供相应的图像。

2.4.1　对病灶的描述　应包含位置、大小、边界、显像剂摄取情况及相应的同机CT所见或其他近期解剖影像所见。病灶显像剂摄取情况可以视觉判断方式或半定量方式（以SUV值表示，其中至少包含最大SUV值）进行描述。病灶大小的测量可使用单个径线（注明短径或长径）或2~3个相互垂直径线描述，注意单位统一。对于随访患者，应注意显像剂摄取和病灶大小的测量方法与前次显像一致性。对于PET/CT检查前其他影像检查（如CT、MRI、超声）发现的病变（包括日期）应描述与之相对应的PET影像所见。

2.4.2　对附加图像的要求　报告中的主要异常所见均需附加相应的截图，并加以必要的标示和文字说明。所给图像应清晰显示病灶的影像特征性表现，与影像所见中的文字表述一致。

2.5　影像诊断意见　鉴于临床医师常习惯于首先阅读PET/CT报告的检查意见，故此部分在报告中最为重要。PET/CT检查意见的关键在于清晰简要，层次分明，避免重复影像所见或进行赘述讨论。要求如下：①影像诊断意见应按照临床诊疗意义排序，首先回答临床主要关注问题，如“是否发现了恶性病变”“病变累及范围及分期”“治疗后随访病变PET及CT的响应评估”等；对于不能明确诊断的病变提出鉴别诊断，并尽可能提出帮助明确病变性质的诊疗措施（如提示适宜的活检部位或有针对性的其他检查方法）。②影像诊断意见所使用的语言应尽可能清晰和明确，如“未见”“可排除”或“考虑为XX疾病”等确定性语言，以避免造成误解。③复诊PET/CT影像诊断应与之前检查（注明日期）对照，提出总体病变数目、大小及代谢的变化情况，并尽可能给出评估意见。

3　报告的签发

报告的书写人员首先应具备相应的执业资质，报告医师完成报告后应仔细检查报告中的所有文字及图像并签字/签章。报告审核是保证医疗质量的有效措施，审核重点在于报告的正确性与合理性，建议双审核，原则上由具有副高级职称及以上的核医学专业医师完成并签字/签章。当诊断困难时，报告审核医师有责任通过与临床进一步沟通了解患者的临床情况，复习相关疾病知识，并组织集体阅片，以保证报告的整体质量。

4　报告范例

PET/CT报告虽无固定的写法，不同中心也有各自的格式，但总体要求一致：即PET/CT的书写要做到客观全面、条理清晰、重点突出、逻辑性强、文字凝练、术语准确、解决临床实际问题。为使广大核医学医师在实际工作中有经验可以借鉴，特附PET/CT报告范例（图1、2）。

(C)1994-2023 China Academic Journal Electronic Publishing House. All rights reserved.　http://www.cnki.net

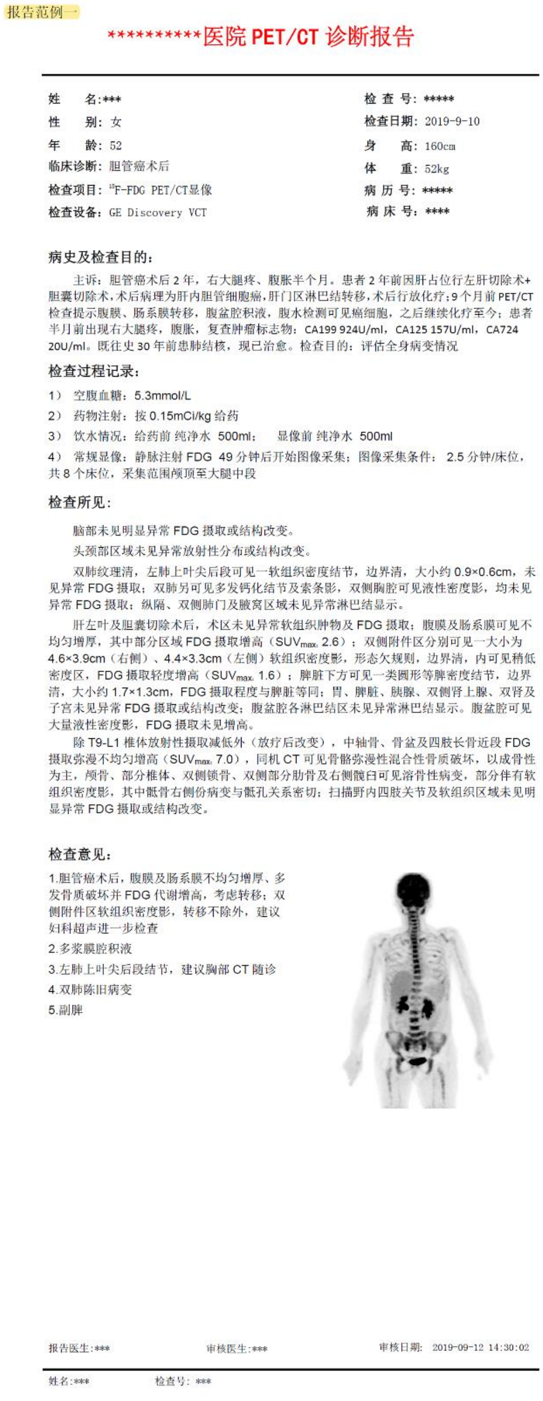

报告范例一

**********医院 PET/CT 诊断报告

姓　　名:***　　　　检 查 号: *****
性　　别: 女　　　　检查日期: 2019-9-10
年　　龄: 52　　　　身　　高: 160cm
临床诊断: 胆管癌术后　　　　体　　重: 52kg
检查项目: ^{18}F-FDG PET/CT显像　　　　病 历 号: *****
检查设备: GE Discovery VCT　　　　病 床 号: ****

病史及检查目的:

主诉: 胆管癌术后 2 年，右大腿疼、腹胀半个月。患者 2 年前因肝占位行左肝切除术+胆囊切除术，术后病理为肝内胆管细胞癌，肝门区淋巴结转移，术后行放化疗；9 个月前 PET/CT 检查提示腹膜、肠系膜转移，腹盆腔积液，腹水检测可见癌细胞，之后继续化疗至今；患者半月前出现右大腿疼，腹胀，复查肿瘤标志物: CA199 924U/ml，CA125 157U/ml，CA724 20U/ml。既往史 30 年前患肺结核，现已治愈。检查目的: 评估全身病变情况

检查过程记录:

1） 空腹血糖: 5.3mmol/L
2） 药物注射: 按 0.15mCi/kg 给药
3） 饮水情况: 给药前 纯净水 500ml;　　显像前 纯净水 500ml
4） 常规显像: 静脉注射 FDG 49 分钟后开始图像采集; 图像采集条件: 2.5 分钟/床位，共 8 个床位，采集范围颅顶至大腿中段

检查所见:

脑部未见明显异常 FDG 摄取或结构改变。

头颈部区域未见异常放射性分布或结构改变。

双肺纹理清，左肺上叶尖后段可见一软组织密度结节，边界清，大小约 0.9×0.6cm，未见异常 FDG 摄取; 双肺另可见多发钙化结节及索条影，双侧胸腔可见液性密度影，均未见异常 FDG 摄取; 纵隔、双侧肺门及腋窝区域未见异常淋巴结显示。

肝左叶及胆囊切除术后，术区未见异常软组织肿物及 FDG 摄取; 腹膜及肠系膜可见不均匀增厚，其中部分区域 FDG 摄取增高（SUV_{max} 2.6）; 双侧附件区分别可见一大小为 4.6×3.9cm（右侧）、4.4×3.3cm（左侧）软组织密度影，形态欠规则，边界清，内可见稍低密度区，FDG 摄取轻度增高（SUV_{max} 1.6）; 脾脏下方可见一类圆形等脾密度结节，边界清，大小约 1.7×1.3cm，FDG 摄取程度与脾脏等同; 胃、脾脏、胰腺、双侧肾上腺、双肾及子宫未见异常 FDG 摄取或结构改变; 腹盆腔各淋巴结区未见异常淋巴结显示。腹盆腔可见大量液性密度影，FDG 摄取未见增高。

除 T9-L1 椎体放射性摄取减低外（放疗后改变），中轴骨、骨盆及四肢长骨近段 FDG 摄取弥漫不均匀增高（SUV_{max} 7.0），同机 CT 可见骨骼弥漫性混合性骨质破坏，以成骨性为主，颅骨、部分椎体、双侧锁骨、双侧部分肋骨及右侧髋臼可见溶骨性病变，部分伴有软组织密度影，其中骶骨右侧份病变与骶孔关系密切; 扫描野内四肢关节及软组织区域未见明显异常 FDG 摄取或结构改变。

检查意见:

1.胆管癌术后，腹膜及肠系膜不均匀增厚、多发骨质破坏并 FDG 代谢增高，考虑转移; 双侧附件区软组织密度影，转移不除外，建议妇科超声进一步检查
2.多浆膜腔积液
3.左肺上叶尖后段结节，建议胸部 CT 随诊
4.双肺陈旧病变
5.副脾

报告医生:***　　审核医生:***　　审核日期: 2019-09-12 14:30:02

姓名:***　　检查号: ***

图 1　报告范例一

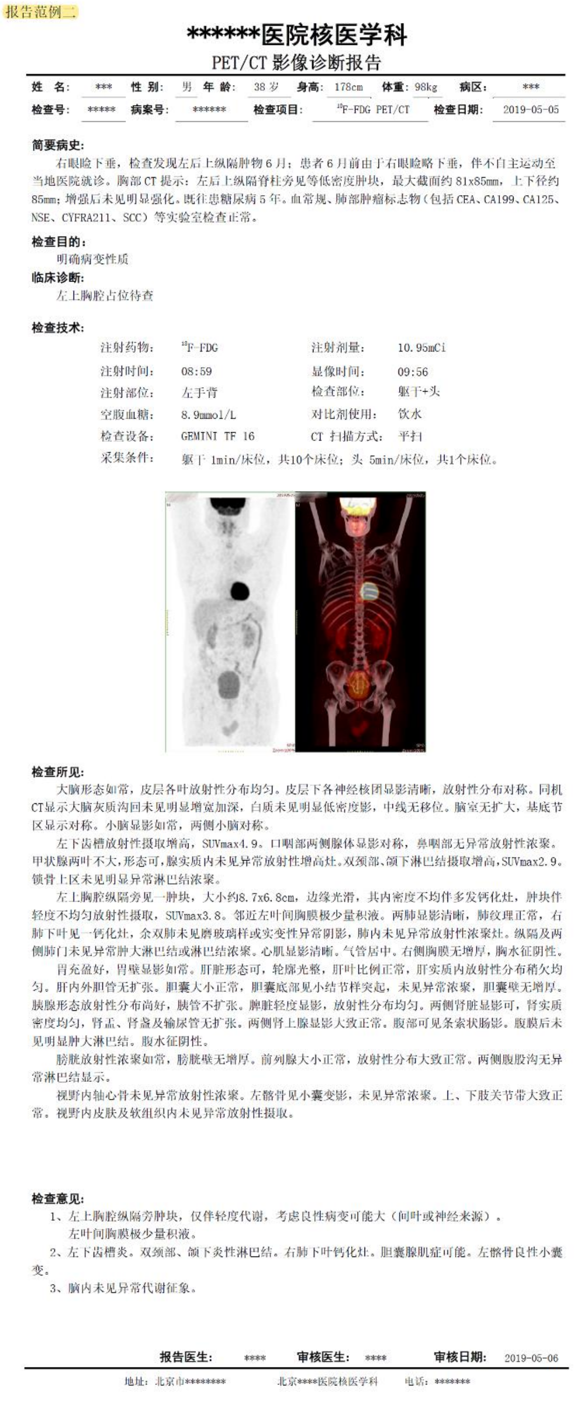

报告范例二

******医院核医学科
PET/CT 影像诊断报告

姓　名: *** 性 别: 男 年 龄: 38 岁 身高: 178cm 体重: 98kg 病区: ***
检查号: ***** 病案号: ****** 检查项目: ^{18}F-FDG PET/CT 检查日期: 2019-05-05

简要病史:

右眼睑下垂，检查发现左后上纵隔肿物 6 月; 患者 6 月前由于右眼睑略下垂，伴不自主运动至当地医院就诊。胸部 CT 提示: 左后上纵隔脊柱旁见等低密度肿块，最大截面约 81x85mm，上下径约 85mm; 增强后未见明显强化。既往患糖尿病 5 年。血常规、肺部肿瘤标志物（包括 CEA、CA199、CA125、NSE、CYFRA211、SCC）等实验室检查正常。

检查目的:

明确病变性质

临床诊断:

左上胸腔占位待查

检查技术:

注射药物: ^{18}F-FDG　　注射剂量: 10.95mCi
注射时间: 08:59　　显像时间: 09:56
注射部位: 左手背　　检查部位: 躯干+头
空腹血糖: 8.9mmol/L　　对比剂使用: 饮水
检查设备: GEMINI TF 16　　CT 扫描方式: 平扫
采集条件: 躯干 1min/床位，共10个床位; 头 5min/床位，共1个床位。

检查所见:

大脑形态如常，皮层各叶放射性分布均匀。皮层下各神经核团显影清晰，放射性分布对称。同机CT显示大脑灰质沟回未见明显增宽加深，白质未见明显低密度影，中线无移位。脑室无扩大，基底节区显示对称。小脑显影如常，两侧小脑对称。

左下齿槽放射性摄取增高，SUVmax4.9。口咽部两侧腺体显影对称，鼻咽部无异常放射性浓聚。甲状腺两叶不大，形态可，腺实质内未见异常放射性增高灶。双颈部、颌下淋巴结摄取增高，SUVmax2.9。锁骨上区未见明显异常淋巴结浓聚。

左上胸腔纵隔旁见一肿块，大小约8.7x6.8cm，边缘光滑，其内密度不均伴多发钙化灶，肿块伴轻度不均匀放射性摄取，SUVmax3.8。邻近左叶间胸膜极少量积液。两肺显影清晰，肺纹理正常，右肺下叶见一钙化灶，余双肺未见磨玻璃样或实变性异常阴影，肺内未见异常放射性浓聚灶。纵隔及两侧肺门未见异常肿大淋巴结或淋巴结浓聚。心肌显影清晰。气管居中。右侧胸膜无增厚，胸水征阴性。

胃充盈好，胃壁显影如常。肝脏形态可，轮廓光整，肝叶比例正常，肝实质内放射性分布稍欠均匀。肝内外胆管无扩张。胆囊大小正常，胆囊底部见小结节样突起，未见异常浓聚，胆囊壁无增厚。胰腺形态放射性分布尚好，胰管不扩张。脾脏轻度显影，放射性分布均匀。两侧肾脏显影可，肾实质密度均匀，肾盂、肾盏及输尿管无扩张。两侧肾上腺显影大致正常。腹部可见条索状肠影。腹膜后未见明显肿大淋巴结。腹水征阴性。

膀胱放射性浓聚如常，膀胱壁无增厚。前列腺大小正常，放射性分布大致正常。两侧腹股沟无异常淋巴结显示。

视野内轴心骨未见异常放射性浓聚。左髂骨见小囊变影，未见异常浓聚。上、下肢关节带大致正常。视野内皮肤及软组织内未见异常放射性摄取。

检查意见:

1、左上胸腔纵隔旁肿块，仅伴轻度代谢，考虑良性病变可能大（间叶或神经来源）。
左叶间胸膜极少量积液。
2、左下齿槽炎。双颈部、颌下炎性淋巴结。右肺下叶钙化灶。胆囊腺肌症可能。左髂骨良性小囊变。
3、脑内未见异常代谢征象。

报告医生: ****　　审核医生: ****　　审核日期: 2019-05-06

地址: 北京市********　　北京****医院核医学科　　电话: *******

图 2　报告范例二

参考文献

[1] Boellaard R, Delgado-Bolton R, Oyen WJG, et al. FDG PET/CT: EANM procedure guidelines for tumour imaging: version 2.0. Eur J Nucl Med Mol Imaging, 2015, 42(2): 328-354.

[2] Niederkohr RD, Greenspan BS, Prior JO, et al. Reporting guidance for oncologic ^{18}F-FDG PET/CT imaging. J Nucl Med, 2013, 54(5): 756-761.

[3] Jamar F, Buscombe J, Chiti A, et al. EANM/SNMMI guideline for 18F-FDG use in inflammation and infection. J Nucl Med, 2013, 54(4): 647-658.

[4] Li YM, Wang Q, Wang XM, et al. Expert consensus on clinical application of FDG PET/CT in infection and inflammation. Ann Nucl Med, 2020, 34(5): 369-376.

【收稿日期】2020-12-25　【修回日期】2021-01-06

（本文编辑　张春辉）

(C)1994-2023 China Academic Journal Electronic Publishing House. All rights reserved.　http://www.cnki.net
